# CX3CR1 deficiency aggravates amyloid driven neuronal pathology and cognitive decline in Alzheimer’s disease

**DOI:** 10.1186/s13024-022-00545-9

**Published:** 2022-06-28

**Authors:** Shweta S. Puntambekar, Miguel Moutinho, Peter Bor-Chian Lin, Vaishnavi Jadhav, Danika Tumbleson-Brink, Ananya Balaji, Martin Alvarado Benito, Guixiang Xu, Adrian Oblak, Cristian A. Lasagna-Reeves, Gary E. Landreth, Bruce T. Lamb

**Affiliations:** 1 Stark Neurosciences Research Institute, Indiana University-School of Medicine, Indianapolis, IN USA; 2 Department of Medical and Molecular Genetics, Indiana University-School of Medicine, Indianapolis, IN USA; 3 Department of Anatomy, Cell Biology and Physiology, Indiana University-School of Medicine, Indianapolis, IN USA; 4 Indiana Biomedical Gateway (IBMG) Program, Indiana University-School of Medicine, Indianapolis, IN USA; 5 Department of Medicine, Division of Gerontology and Geriatric Medicine, University of Washington, Seattle, WA USA; 6 Indiana Clinical and Translational Institute (CTSI), Summer Research Program (SRP), Indianapolis, IN USA; 7 Department of Radiology, Indiana University-School of Medicine, Indianapolis, IN USA

**Keywords:** Amyloid, CX3CR1, Microglia, Neurodegeneration, Tau

## Abstract

**Background:**

Despite its identification as a key checkpoint regulator of microglial activation in Alzheimer’s disease, the overarching role of CX3CR1 signaling in modulating mechanisms of Aβ driven neurodegeneration, including accumulation of hyperphosphorylated tau is not well understood.

**Methodology:**

Accumulation of soluble and insoluble Aβ species, microglial activation, synaptic dysregulation, and neurodegeneration is investigated in 4- and 6-month old 5xFAD;*Cx3cr1*^+*/*+^ and 5xFAD;*Cx3cr1*^*−/−*^ mice using immunohistochemistry, western blotting, transcriptomic and quantitative real time PCR analyses of purified microglia. Flow cytometry based, *in-vivo* Aβ uptake assays are used for characterization of the effects of CX3CR1-signaling on microglial phagocytosis and lysosomal acidification as indicators of clearance of methoxy-X-04^+^ fibrillar Aβ. Lastly, we use Y-maze testing to analyze the effects of *Cx3cr1* deficiency on working memory.

**Results:**

Disease progression in 5xFAD;*Cx3cr1*^*−/−*^ mice is characterized by increased deposition of filamentous plaques that display defective microglial plaque engagement. Microglial Aβ phagocytosis and lysosomal acidification in 5xFAD;*Cx3cr1*^*−/−*^ mice is impaired *in-vivo*. Interestingly, *Cx3cr1* deficiency results in heighted accumulation of neurotoxic, oligomeric Aβ, along with severe neuritic dystrophy, preferential loss of post-synaptic densities, exacerbated tau pathology, neuronal loss and cognitive impairment. Transcriptomic analyses using cortical RNA, coupled with qRT-PCR using purified microglia from 6 month-old mice indicate dysregulated TGFβ-signaling and heightened ROS metabolism in 5xFAD;*Cx3cr1*^*−/−*^ mice. Lastly, microglia in 6 month-old 5xFAD;*Cx3cr1*^*−/−*^ mice express a ‘degenerative’ phenotype characterized by increased levels of *Ccl2*, *Ccl5*, *Il-1β*, *Pten* and *Cybb* along with reduced *Tnf*, *Il-6* and *Tgfβ1* mRNA.

**Conclusions:**

*Cx3cr1* deficiency impairs microglial uptake and degradation of fibrillar Aβ, thereby triggering increased accumulation of neurotoxic Aβ species. Furthermore, loss of *Cx3cr1* results in microglial dysfunction typified by dampened TGFβ-signaling, increased oxidative stress responses and dysregulated pro-inflammatory activation. Our results indicate that Aβ-driven microglial dysfunction in *Cx3cr1*^*−/−*^ mice aggravates tau hyperphosphorylation, neurodegeneration, synaptic dysregulation and impairs working memory.

**Supplementary information:**

The online version contains supplementary material available at 10.1186/s13024-022-00545-9.

## Background

Neuroinflammation and glial activation in Alzheimer’s disease (AD) precede the onset of extracellular β-amyloid (Aβ) plaque deposition and evolve during the development of neurofibrillary tangles (NFTs) and neurodegeneration. These underlying signaling pathways are postulated to lay the framework for cognitive impairment seen in AD [1]. Single-nuclei RNA sequencing (snRNA-seq) data have indicated that distinct clusters of activated microglia are associated with Aβ vs. NFTs vs. inflammatory signaling in AD [2, 3], and molecular networks between these unique disease-associated glial clusters can reciprocally shape their activation profiles [4]. Lastly, large scale GWAS studies identifying single-nucleotide polymorphisms (SNPs) proximal to microglial-enriched, innate immune genes like *CR1*, *CD33*, *MEF2C*, *HLA-DRB5-DRB1, PTK2b* and *TREM2* that significantly increase the risk of AD [5] have further underscored the critical role of microglia in neurodegeneration and disease progression.

Recently, loss-of-function variants in the microglial fractalkine receptor (CX3CR1) have been associated with worsened Braak staging in AD and neurodegeneration along with reduced survival in amyotrophic lateral sclerosis (ALS), implicating these SNPs as disease-modifying variants in neurodegenerative diseases [6–8]. Signaling via CX3CR1 and its neuronal ligand CX3CL1 represents one of multiple important neuro-glial communication axes that maintain microglial homeostasis [9, 10]. *Cx3cr1* deficiency is associated with a transient deficit in microglial abundance during early post-natal development [11, 12]. This correlates with reduced synaptic engulfment and deficits in synapse maturation and elimination during early post-natal development [11, 12], resulting in decreased functional brain connectivity, impaired social-interaction and increased autism-like, repetitive behaviors in adult mice [11]. CX3CR1-signaling not only actively dampens microglial phagocytosis and neurotoxic/pro-inflammatory activation during healthy aging, but also inhibits NMDA and glutamate dependent Ca^2+^ influx into neurons, thereby protecting against neuronal excitotoxicity [13–15]. Indeed, disruption of *Cx3cr1* increases pro-inflammatory microglial signaling that correlates with heightened loss of dopaminergic neurons in the substantia nigra in murine models of Parkinson’s disease [16, 17]. Similar results have been reported in the SOD1-G93A model of ALS, where *Cx3cr1* deficiency is associated with increased SOD1 aggregation, neuronal loss and inflammatory microglial activation with impaired autophagy-lysosomal degradation pathways and autophagosome maturation [18, 19]. These studies highlight the context-dependent nature of neuroprotective, microglial CX3CR1 signaling.

Single-cell RNA sequencing (scRNA-seq) studies have suggested that downregulation of *Cx3cr1* in plaque-associated microglia is a primary event in the neuroinflammatory cascade in neurodegenerative diseases [20–22]. Attenuated *Cx3cr1* expression represents a shift in microglia towards a protective phenotype associated with increased expression of *Trem2*, *Apoe*, *CD68*, *Axl*, *MerTK* and *Lpl*, indicative of enhanced capacity for TREM2-dependent plaque compaction, Aβ phagocytosis and lipid metabolism [20]. In contrast, activation of TREM2-APOE signaling in AD results in a subset of microglia that display a ‘neurodegenerative phenotype’, characterized by suppression of homeostatic genes like *Cx3cr1* and *P2ry12* and upregulation of pro-inflammatory markers like *Clec7a*, *Ccl5*, *Ccl2*, *IL1b*, and *Nos2*. These neurodegenerative microglia are associated with neuritic plaques, degenerating neurons and trigger a loss of tolerogenic responses in EAE and ALS [21]. Thus, downregulation of *Cx3cr1* has distinct effects on plaque clearance and subsequent neurotoxicity in AD.

Our previous studies using murine models of AD deficient in *Cx3cr1* have provided insights into the divergent role of fractalkine signaling in amyloidosis and tauopathy [23–25]. In the APPPS1 and R1.40 mouse models of amyloidosis, *Cx3cr1* deficiency results in a gene-dose dependent reduction in fibrillar Aβ (fAβ) burdens in *early* disease, suggesting that a loss of CX3CR1 is beneficial in Aβ pathogenesis. Interestingly, 4 month-old APPPS1;*Cx3cr1*^*−/−*^ mice display impaired microglial plaque engulfment with altered activation of plaque-associated microglia as evidenced by reduced CD68 and CD45 immunoreactivity [25]. By contrast, in the hTau model of tauopathy, loss of *Cx3cr1* enhances microglial activation, increases accumulation of Gallays^+^ neuronal NFTs and exacerbates cognitive dysfunction [23]. Furthermore, reactive microglia in hTau;*Cx3cr1*^*−/−*^ mice drive the spread of pathological pTau, possibly via the IL-1β signaling pathway [24]. Overall, our data suggests that while the loss of microglial CX3CR1 may enhance plaque clearance in early stages of AD, it may aggravate long term neurodegeneration. However, given that pathological Aβ is the primary trigger of the neuropathological cascade in AD, insights on how Aβ-triggered microglial activation shapes subsequent plaque associated neurotoxicity and how CX3CR1 signaling affects these pathways are still lacking.

In this report, we investigate how microglial responses to Aβ shaped by CX3CR1 drive long term neurotoxicity in the 5xFAD model of amyloid disease. Our results indicate that *Cx3cr1* deficiency exacerbates neurodegeneration and cognitive impairment with disease progression in 5xFAD animals by driving increased accumulation of neurotoxic oligomeric Aβ (oAβ), fibrillar Aβ (fAβ) plaques, intraneuronal inclusions of hyperphosphorylated tau (pTau) and enlarged foci of neuritic dystrophy. These effects are potentiated, in part, due to microglial dysfunction in 5xFAD;*Cx3cr1*^*−/−*^ mice as evidenced by impaired microglial Aβ-phagocytosis and clearance as well as aberrant TGFβ-signaling, inflammatory activation and reactive oxygen species (ROS) metabolism. Lastly, in contrast to 5xFAD;*Cx3cr1*^+*/*+^ mice in which pTau pathology strongly correlates with accumulation of compact plaques and small foci of neuritic dystrophy, aggravated deposition of pathological tau in the absence of *Cx3cr1* is correlated with, oligomeric Aβ, filamentous Aβ and large dystrophic neurites.

## Materials and methods

### Animals

5xFAD mice on the C57BL/6 J background were obtained through Jackson Laboratories in collaboration with the Model-AD Center at the IU School of Medicine (Stock # 34,848-JAX). 5xFAD mice were maintained as hemizygotes for the APP and PSEN1 transgenes (5xFAD^±^). B6.129P2(Cg)-Cx3cr1tm1Litt/J mice on the congenic background were purchased through Jackson Laboratories (*Cx3cr1*^*−/−*^: Stock # 005,582). In these mice, the first 390 base-pairs of the coding exon 2 of the *Cx3cr1* gene are replaced by the enhanced green fluorescent protein (EGFP) sequence, thereby resulting in a loss of *Cx3cr1* gene expression. *Trem2*^*−/−*^ mice on the C57BL/6 J background were purchased through Jackson Laboratories (Stock # 027,197) and mated with 5xFAD^±^ mice to generate 5xFAD;*TREM2*^*−/−*^ mice. 5xFAD^±^ mice were mated with *Cx3cr1*^*−/−*^ animals, and progeny were subsequently intercrossed to generate 5xFAD^±^;*Cx3cr1*^*−/−*^ cohorts (henceforth referred to as 5xFAD;*Cx3cr1*^*−/−*^). Pathology in the absence of *Cx3cr1* was compared to age-matched 5xFAD^±^;*Cx3cr1*^+*/*+^ (henceforth referred to as 5xFAD;*Cx3cr1*^+*/*+^) mice throughout the study. Age-matched littermates that do not express 5xFAD mutations (C57BL/6 J;5xFAD^−/−^, henceforth referred to as B6) were used as controls throughout. All animals were housed in animal facilities within Stark Neurosciences Research Institute (SNRI) at The Indiana University School of Medicine (IUSM), accredited by the Association and Accreditation of Laboratory Animal Care. Animals were maintained according to USDA standards and the National Institutes of Health Guide for the Care and Use of Laboratory Animals. All experiments were approved by the IUSM Institutional Animal Care and Use Committee.

### Immunohistochemistry

Three Male and 3 female mice of each genotype were used for all histochemical analyses, based on power analyses for an 80% probability of detecting a 25% change in AD pathological outcomes, based on previous publications [25–27]. Brain tissues fixed in 4% PFA were cryoprotected in 30% sucrose and embedded in OCT. Embedded brains were processed into free-floating, 30µms thick, sagittal sections using a Leica Cryostat and stored at -20ºC in cryostorage solution. Immunofluorescence staining was done as previously described [25]. Briefly, sections were washed with 1XPBS and incubated with a 10 mM sodium citrate solution containing tween-20 (pH = 6.00) for 15 min, at 90ºC to quench microglial EGFP. Sections were cooled for 30 min at room temperature (RT) and incubated in a blocking solution containing 5% normal donkey serum (Sigma Aldrich; Cat # D9663-10ML) and 0.5% Triton-X100 (Sigma Aldrich; Cat # X100-100ML) for 1 h at RT. Mouse on Mouse (MOM) Blocking Reagent (1:1000, Vector Laboratories) was added to the blocking solution if primary antibodies used were generated in mouse or rat. Tissues were incubated overnight at 4ºC in blocking solution with primary antibodies, followed by incubation species-specific Alexa-fluor conjugated secondary antibodies (1:1000, Life Technologies). Sections were mounted on Superfrost Plus glass slides and air-dried for 30–45 min. For visualization of fibrillar Aβ_42_ plaques, slides were dipped in a 1% Thioflavin-S (ThioS) solution, de-stained with 70% ethanol and washed with 1XPBS prior to being coverslipped with ProlongGold.

For 3,3′-diaminobenzidine (DAB) staining, sections were incubated in a solution of 1% H_2_O_2_ in 1XPBS for 30 min at RT to quench endogenous peroxidases. Antigen retrieval was performed by incubating sections in 10 mM sodium citrate (pH = 6.00) at 90ºC for 10 min. Sections were incubated in blocking solution containing 5% normal goat serum, 0.5% Triton-X100 and Mouse-On-Mouse (M.O.M) blocking reagent (1:1000) for 1 h at RT. Tissues were incubated overnight at 4ºC in blocking buffer with Mouse α-AT8. Following incubation with biotinylated α-mouse IgG (1:200, Invitrogen, Cat # B-2763), slices were developed using the VECTASTAIN Elite ABC kit (Vector Laboratories) and DAB. Sections were mounted and coverslipped using Permount (FisherScientific, Cat # SP15-100). Details for all primary antibodies used for immune-fluorescent and DAB staining are listed in Supplementary Table 1.

### Imaging and analysis

High resolution fluorescence imaging was done using the Nikon AR1 confocal microscope. High-resolution, brightfield images were acquired using the CTR5000 upright Leica microscope. Post processing and analysis was done using ImageJ (National Institutes of Health). Branching and junction analysis for Iba1^+^ microglia in B6;*Cx3cr1*^+*/*+^ and B6;*Cx3cr1*^*−/−*^ mice was done using the ‘Skeletonize’ and ‘Analyze Skeleton’ plugins in ImageJ. For analysis of plaque circularity and Iba1 occupancy, 15–20 μm Z-stacks were imaged at a 60X magnification. Circularity analysis on ThioS^+^ plaques was done using the ‘Shape Descriptors’ plugin in ImageJ. Cut offs for plaque circularity were defined as previously published by Yuan P. et al. [28], where filamentous plaques had a circularity score of 0.00–0.14 and compact plaques had circularities greater than 0.30. Plaques with circularity scores between 0.15–0.28 were classified as having ‘intermediate’ phenotypes. To quantify microglial process-engagement with ThioS^+^ plaques, regions-of-interest (ROIs) were traced along ThioS^+^ plaque borders in serial sections co-stained with ThioS and Iba1. Defined ROIs were applied to the Iba1 layer, and the percentage of area within the ROI positive for Iba1 immunoreactivity was quantified. Neuronal numbers within the subiculum were counted automatically using the particle analysis feature. The watershed plugin was used for segmentation of tightly packed NeuN^+^ cell bodies. Dystrophic neurites were counted manually. For size classification, the line selection tool was used to manually threshold out particles less than 500 µm (50-500 µm), followed by subtraction of the number of dystrophic neurites > 500 μm (500-1000 µm) from the total number of dystrophic neurites.

### Flow cytometry based in-vivo Aβ phagocytosis assay and FACS-based microglial purification

fAβ internalized by resident microglia was analyzed using methoxy-X04, a brain-penetrant dye that specifically binds to fibrillar β-sheet deposits [29]. Briefly, cohorts of B6;*Cx3cr1*^+*/*+^, B6;*Cx3cr1*^*−/−*^, 5xFAD;*Cx3cr1*^+*/*+^ *and* 5xFAD;*Cx3cr1*^*−/−*^ were injected intraperitoneally (i.p.) with 10 mg/kg of methoxy-X04 and sacrificed 3 h post injection. Brain-derived mononuclear cells were prepared as previously described [30]. Percoll-derived mononuclear cells were resuspended in FACS buffer (0.25% BSA in 1XPBS), incubated with rat anti-mouse FcγIII/II MAb (BD Biosciences, Cat # 2.4G2) to eliminate non-specific antibody binding and stained with phycoerythrin-cyanin7 (PeCy7)- or phycoerythrin (PE)-conjugated antibodies specific for CD11b (M1/20) and CD45 (30-F11). Microglia were defined based on their CD11b^+^CD45^low^ vs CD11b^+^CD45^high^ profiles. To assess endolytic activation, cells were incubated with 20 nM Lysotracker™ DeepRed (Life Technologies, Cat # L12492). Lysotracker™ DeepRed and methoxy-X04 were detected using the 633 nm and 421 nm lasers respectively. All experiments were done using appropriate single-colored, compensation controls to eliminate any non-specific bleed-through due to spectral overlap.

For microglial purification, percoll-derived mononuclear cells, resuspended in FACS buffer were Fc-blocked with rat anti-mouse FcγIII/II MAb (BD Biosciences, Cat # 2.4G2) and incubated with PE-conjugated α-CD11b (M1/20) antibodies. CD11b^+^ microglial cells were purified using the Sony MA900 cell sorter. FACS purified cell populations were recovered by centrifugation at 400 × g for 7 min at 4 °C, resuspended in TRIzol (Invitrogen, Carlsbad, CA) and stored at − 80 °C for subsequent RNA extraction and cDNA synthesis. Cell yields ranged between 400,000–600,000 CD11b^+^ microglia per brain.

### RNA extraction, cDNA synthesis and quantitative Real Time PCR (qRT-PCR)

150-200µls of homogenized cortical lysates were mixed with an equal volume of QIAzol lysis reagent (QIAGEN RNeasy kits). RNA was isolated using QIAGEN RNeasy kit as per the manufacturer’s protocol and quantified using NanoDrop (Thermo Scientific). 1 µg RNA was used to synthesize cDNA using the High Capacity RNA-to-cDNA kit (Applied Biosystems). Cellular TRIzol fractions were treated with chloroform, and RNA was precipitated with 2-propanol, washed with 75% ethanol and resuspended in RNAse free water. Following DNAse treatment using the DNA Free™ kit (Ambion), cDNA was synthesized using the Moloney murine leukemia virus (MMLV) reverse transcriptase kit (Invitrogen). Gene expression was normalized to *Gapdh* (for qRT-PCRs using FACS purified microglia) or *Hprt* (for qRT-PCRs using cortical cDNA) and expressed as fold changes relative to microglia isolated from 5xFAD;*Cx3cr1*^+*/*+^ females (for qRT-PCRs using FACS purified microglia) or B6;*Cx3cr1*^+*/*+^ mice (for qRT-PCRs using cortical cDNA) following the 2^−ΔΔCt^ method, where ΔΔCt = ΔCt (Target sample) – ΔCt (Reference sample). Taqman probes used are as follows – Microglial activation: Purinergic receptor P2Y12 (*P2ry12*: Mm01289605_m1), Itgam (*Cd11b*: Mm00434455_m1), Ionized calcium binding adaptor molecule-1 (*Iba1*: Mm00479862_g1); *Spi1/Pu.1*: Mm00488140_m1, Apolipoprotein E (*Apoe*; Mm01307192_m1), C-Type Lectin Domain Containing 7A (*Clec7a*; Mm01183349_m1), Cystatin F (*Cst7*; Mm00438349_m1), transforming growth factor β-1 (*Tgfβ1*; Mm01178820_m1), transforming growth factor-β-receptor 1 (*TgfβR1*; Mm00436964_m1). Neuroinflammation: chemokine (C–C motif) ligand 2 (*Ccl2*; Mm00441241_ml), chemokine (C–C motif) ligand 5 (*Ccl5*; Mm01302427_m1), interleukin 1β (*Il-1β*; Mm00434228_ml), interleukin 6 (*Il-6*; Mm00446191_m1), tumor necrosis factor (*Tnf*; Mm00443258_m1). ROS metabolism: phosphatase and tensin homolog (*Pten*; Mm00477208_m1), PTEN-induced kinase-1 (*Pink1*; Mm00550827_m1), cytochrome b-245 beta chain (*Cybb*; Mm01287743_m1).

### Nanostring nCounter Gene expression

The NanoString Mouse Neuroinflammation gene expression panel (Cat # LBL-10492–02) was used for gene expression profiling on the nCounter platform (NanoString, Seattle, WA) as described by the manufacturer. nSolver software was used for analysis of NanoString gene expression values. Normalization to identify differentially expressed genes (DEGs) was done by dividing counts within a lane by geometric mean of the housekeeping genes from the same lane. Gene Ontology analyses for determination of top functional pathways affected in 5xFAD;*Cx3cr1*^*−/−*^ mice with respect to 5xFAD;*Cx3cr1*^+*/*+^ cohorts were done using the Panther Classification System (http://www.pantherdb.org/). KEGG enrichment analyses for determination of top signaling pathways altered in 5xFAD;*Cx3cr1*^*−/−*^ mice were done using the VolcanoR software (http://45.8.90.25:3838/volcanoR/). Statistical differences for this data set were calculated using the Benjamini-Hotchberg method within nSolver, Panther Classification System and VolcanoR software.

## Supplemental materials

### Supplemental data

Supplemental Table 1 lists antibodies used for immunohistochemistry, western blots and flow cytometry, along with their vendor/catalogue information. Supplemental Table 2 lists results for correlation analyses between dystrophic neurites, plaque phenotypes and pTau pathology in cortices of 6 month-old 5xFAD;*Cx3cr1*^+*/*+^ and 5xFAD;*Cx3cr1*^*−/−*^ mice. Supplemental Fig. 1 shows identical microglial phenotypes and numbers in adult B6;*Cx3cr1*^+*/*+^ and B6;*Cx3cr1*^*−/−*^ mice. Supplemental Fig. 2 shows that *Cx3cr1* deficiency does not alter microglial proliferation, microglial recruitment to Aβ plaques and TREM2 levels in the 5xFAD brain. Supplemental Fig. 3  shows GO and KEGG analysis of significant DEGs in transcriptomic profiling of neuroinflammatory changes in cortical lysates of 6 month-old 5xFAD;*Cx3cr1*^+*/*+^ vs. 5xFAD;*Cx3cr1*^*−/−*^ mice. Supplemental Fig. 4 shows similar expression of genes associated with microglial activation signatures, inflammatory activation and oxidative stress response in cortex of 6 month-old B6;*Cx3cr1*^+*/*+^ and B6;*Cx3cr1*^*−/−*^ mice. Supplemental Fig. 5 shows gating strategy to identify fAβ^+^ microglial subsets in B6 and 5xFAD mice. Supplemental Fig. 6 shows flow cytometry analysis of microglial fAβ uptake and microglial endolytic activation in 4 month-old 5xFAD;*Cx3cr1*^+*/*+^ and 5xFAD;*Cx3cr1*^*−/−*^ mice. Supplemental Fig. 7 shows accumulation of larger foci of dystrophic neurites in the cortex of 6 month-old 5xFAD;*Cx3cr1*^*−/−*^ mice. Supplemental Files 8 and 9 are excel sheets for all differentially expressed genes as queried using the Nanostring Neuroinflammation panel for 6 month old female and male 5xFAD;*Cx3cr1*^*−/−*^ vs. 5xFAD;*Cx3cr1*^+*/*+^ mice.

**Fig. 1:**
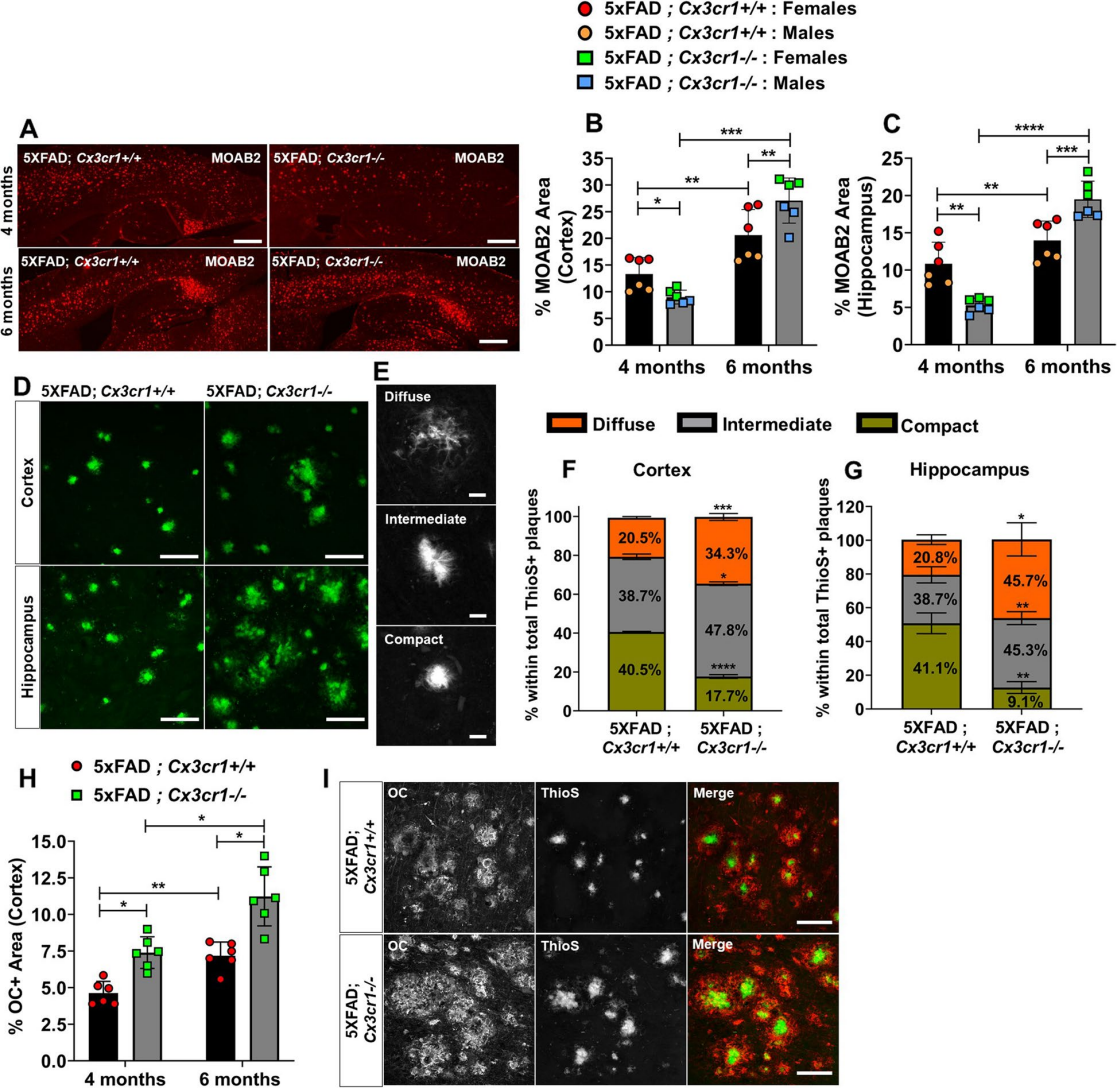
Accelerated plaque deposition in 5xFAD mice deficient in *Cx3cr1*. (**A)** Accumulation of MOAB2^+^ Aβ_42_ plaques in (top panels) 4 month-old vs. (bottom panels) 6 month-old 5xFAD*;Cx3cr1*^+*/*+^ and 5xFAD; *Cx3cr1*^*−/−*^ mice. Scale bars = 500 μm. Quantification of %MOAB2^+^ areas in the (**B)** cortex and (**C)** hippocampus of 4 and 6 month-old 5xFAD;*Cx3cr1*^+*/*+^ (black bars) and 5xFAD;*Cx3cr1*^*−/−*^ (grey bars) mice. Data in B,C represent mean proportions of cortical and hippocampal MOAB2^+^ areas quantified using *n* = 6 animals (3 females, 3 males) per genotype, per time-point. Error bars represent SEM. Statistical analysis done using two-way ANOVA (p^int^ cortex < 0.0001, p^int^ hippocampus < 0.0001) followed by Tukey’s post hoc tests. (**D)** ThioS^+^ plaques visualized in the (top panels) cortex and (bottom panels) hippocampus of 6 month-old 5xFAD mice with and without *Cx3cr1*. Scale bars = 50 µm. (**E)** Identification of morphologically distinct ThioS^+^ plaques based on circularity scores as follows – Diffuse: Circularity = 0.00–0.14, Intermediate: Circularity = 0.15–0.28, Compact: Circularity =  > 0.30. Scale bars = 30 µm. Proportion of ThioS^+^ plaques with diffuse (orange), intermediate (grey) and compact (green) circularities were quantified in the (**F**) cortex (**G**) hippocampus of 6 month-old 5xFAD;*Cx3cr1*^+*/*+^ and 5xFAD;*Cx3cr1*^*−/−*^ mice. Data in F,G represent mean proportions of each plaque type quantified using 6 mice (3 females, 3 males) per genotype at each age. Circularity analysis was based on 250–300 cortical plaques and 100–150 hippocampal plaques per animal, per genotype at each age. Error bars represent SEM. Statistical analysis done using two-way ANOVA (p^int^ cortex and p^int^ hippocampus = 0.0002) followed by Tukey’s post hoc tests. (**H**) Quantification of cortical area occupied by OC^+^ oAβ in 4- and 6-month-old 5xFAD;*Cx3cr1*^+*/*+^ (black bars) and 5xFAD;*Cx3cr1*^*−/−*^ (grey bars) mice. Data represents mean proportion of OC^+^ cortical area quantified using *n* = 6 (3 females, 3 males) of each genotype at each age. Statistical analysis done using two-way ANOVA (p^int^ = 0.03) followed by Tukey’s post hoc tests. (**I**) Accumulation of soluble OC^+^ oAβ around ThioS^+^ plaques in 6 month-old 5xFAD*;Cx3cr1*^+*/*+^ and 5xFAD;*Cx3cr1*.^*−/−*^ mice. Scale bars = 100 μm. *p < 0.01, **p < 0.001, ***p < 0.0001. ****p < 0.00001. All histology data representative of *n* = 6 mice (3 females, 3males) per genotype at each age analyzed

**Fig. 2:**
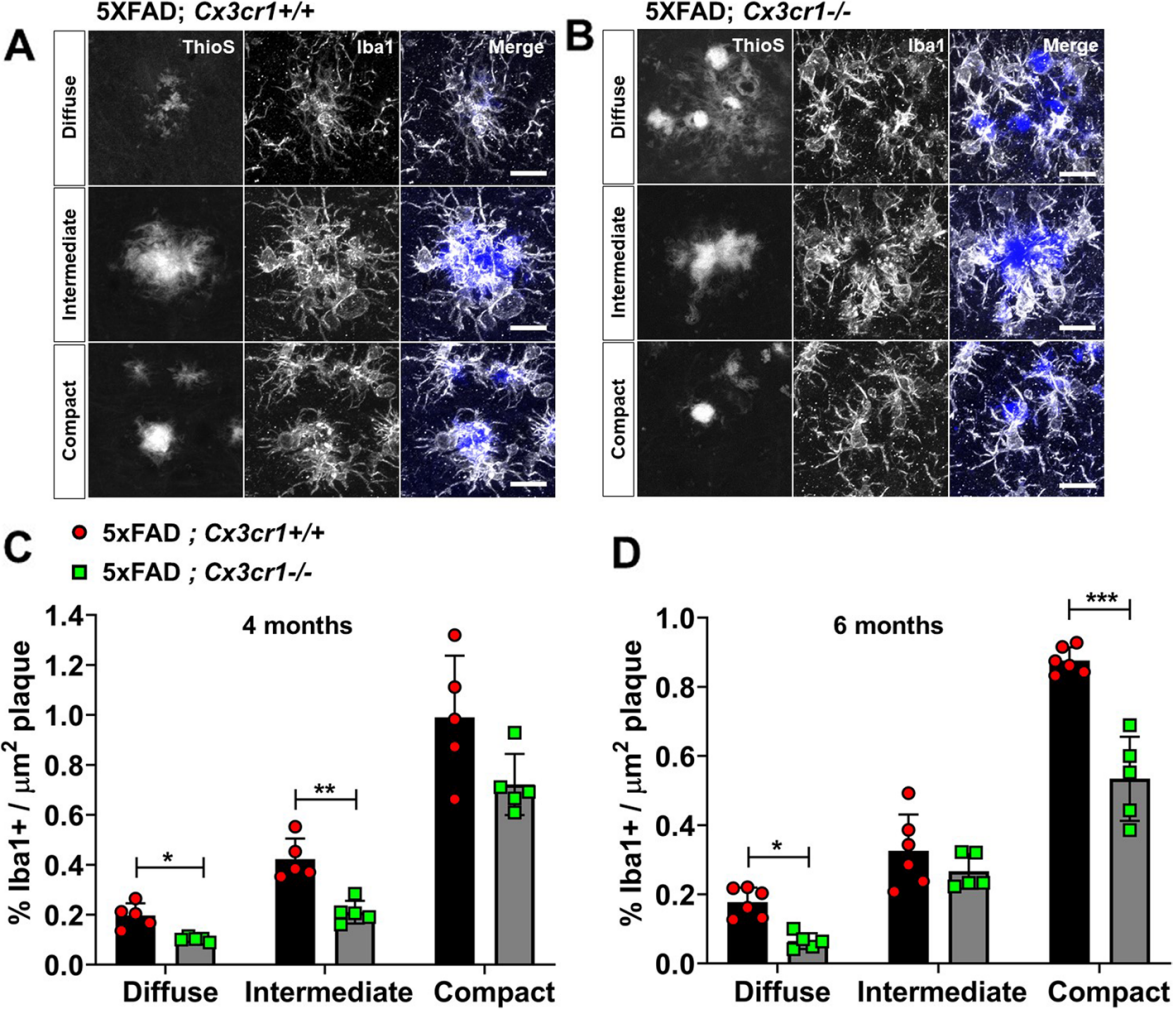
Impaired microglial plaque engagement in 4- and 6-month old 5xFAD mice deficient in *Cx3cr1*. Co-labeling of ThioS^+^ plaques and Iba1^+^ microglia in the cortex of 6 month-old (**A**) 5xFAD;*Cx3cr1*^+*/*+^ and (**B**) 5xFAD;*Cx3cr1*^*−/−*^ mice. Images representative of cortical plaques visualized using 6 mice (3 females, 3 males) per genotype. Scale bars = 30 µm. Iba1 occupancy of the area within regions-of-interest (ROI) traced along the boundaries of diffuse, intermediate, and compact plaques calculated for plaques in the cortex of (**C**) 4 month-old and (**D**) 6 month-old 5xFAD;*Cx3cr1*^+*/*+^ (black bars) and 5xFAD;*Cx3cr1*^*−/−*^ (grey bars) mice. Data in C,D represent the mean %Iba1^+^ area calculated within ROIs defined around cortical plaques in 4- and 6-month old 5xFAD mice with and without *Cx3cr1* (*n* = 5–6 female and male mice of each genotype, per age). Error bars represent SEM. ~ 250–350 plaques were analyzed using multiple sections for each animal/genotype at each age. Statistical analysis done using Two-way ANOVA (p^int^ 4 month < 0.005, p.^int^ 6 month = 0.0002) followed by Sidak’s post hoc tests. ***p < 0.0001, **p < 0.001, *p < 0.01

**Fig. 3:**
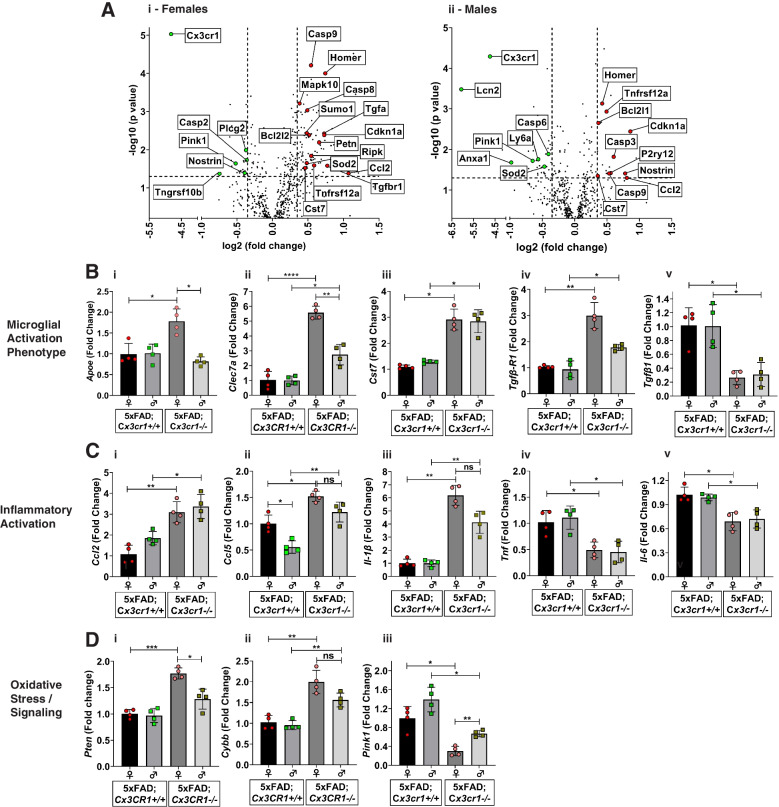
Dysregulation of microglial activation in the absence of *Cx3cr1.* (**A**) Volcano plots showing significant differentially expressed genes (DEGs) identified based on the Nanostring neuroinflammation panel to analyze cortical lysates from (i) 6 month-old female and (ii) 6 month-old male 5xFAD;*Cx3cr1*^*−/−*^ mice when compared to sex-matched 5xFAD;*Cx3cr1*^+*/*+^ animals. (**B-D**) Quantitative real-time PCR (qRT-PCR) analyses performed on CD11b^+^ microglia purified from brains of 6 month-old 5xFAD;*Cx3cr1*^+*/*+^ and 5xFAD;*Cx3cr1*^*−/−*^ mice to validate top DEGs from Nanostring analysis. Gene-expression associated with (Bi-Bv) Disease-associated microglial activation, (Ci-Cv) inflammatory activation and (Di-Diii) oxidative stress responses analyzed using purified microglia from 5xFAD mice with and without *Cx3cr1*. All gene-expression data are represented as fold-change compared to microglia isolated from female 5xFAD;*Cx3cr1*^+*/*+^ mice (black bars). Data in **B-D** represent mean ddCT values using 8 mice (4 females, 4 males) per genotype. Error bars represent SEM. Statistics done One-way ANOVA followed by Brown-Forsythe and Welch post-hoc tests. *p^adj^ < 0.05, **p^adj^ < 0.001, ***p^adj^ < 0.0001, ****p.^adj^ < 0.00001

**Fig. 4:**
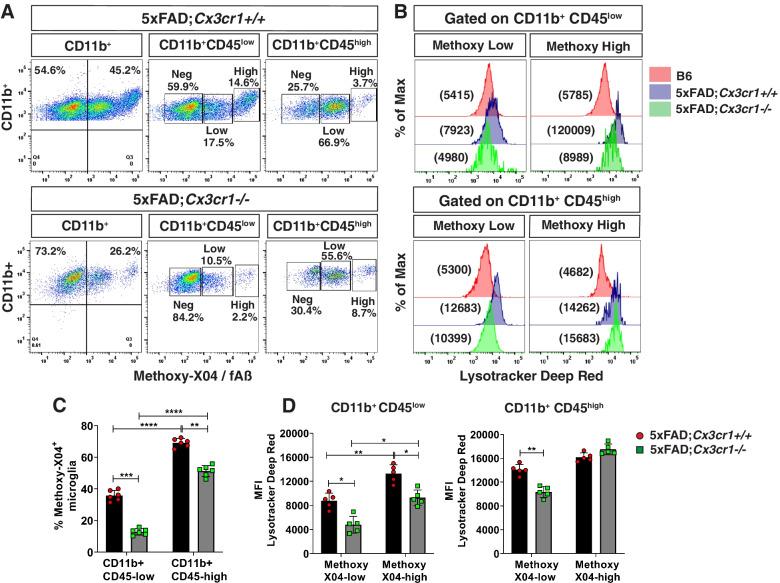
Impaired uptake of fAβ and lysosomal acidification in 6 month-old 5xFAD;*Cx3cr1*^*−/−*^ mice. (**A)** Representative flow cytometry plots identifying subpopulations of methoxy-X04^+^CD11b^+^CD45^low^ and methoxy-X04^+^CD11b^+^CD45^high^ microglia and (**B**) mean fluorescence intensities (MFI) for Lysotracker Deep-Red™ (-DR) to assess the acidification of endolytic compartments of these individual subpopulations. Data representative of flow cytometry experiments done using *n* = 5–6 mice (males and females) of 6 month-old 5xFAD;*Cx3cr1*^+*/*+^ and 5xFAD;*Cx3cr1*^*−/−*^ mice. Quantification of (**C**) proportion of methoxy-X04^+^CD11b^+^CD45^low^ and methoxy-X04^+^CD11b^+^CD45^high^ microglia and (**D**) Lysotracker-DR MFIs for methoxy-X04^low^ and methoxy-X04^high^ microglia within the CD11b^+^CD45^low^ and CD11b^+^CD45^high^ subsets in 6 month-old 5xFAD;*Cx3cr1*^+*/*+^ (black bars) and 5xFAD;*Cx3cr1*.^*−/−*^ (grey bars) mice. Data in C,D represents means calculated using *n* = 5–6 mice (males and females) per genotype. Error bars represent SEM. All animals were processed and analyzed in a single experiment to enable MFI comparisons across genotypes and individual mice. Statistics for data in C,D was done using two-way ANOVA with Tukey’s multiple comparison tests. *p < 0.05, **p < 0.001, ***p < 0.0001, ****p < 0.00001

**Fig. 5:**
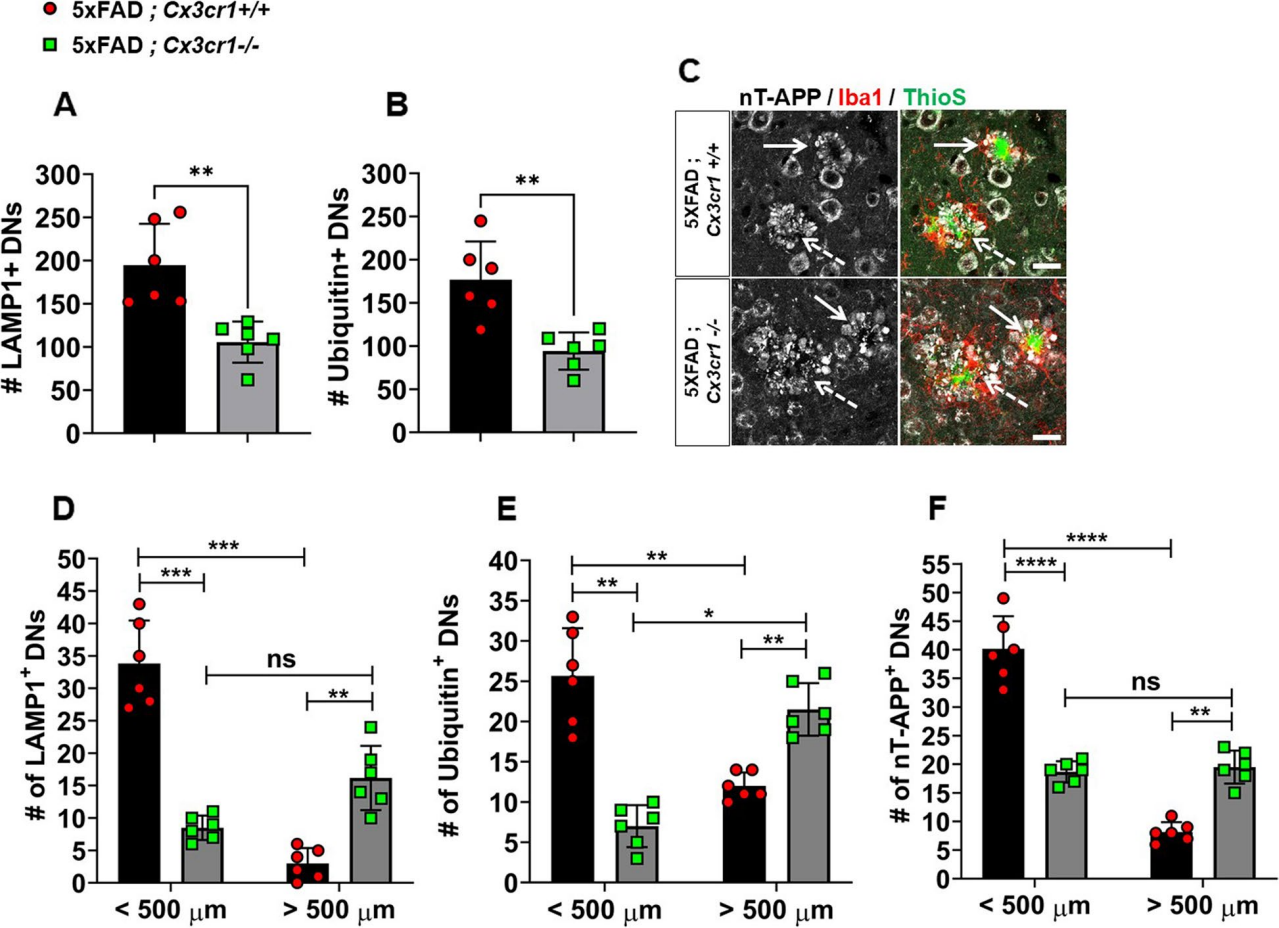
Early accumulation of larger foci of severe neuritic dystrophy in 4 month-old 5xFAD;*Cx3cr1*^*−/−*^ mice. Characterization of cortical dystrophic neurites (DNs) in 4 month-old 5xFAD mice with and without *Cx3cr1* using α-LAMP1, α-Ubiquitin and α-nT-APP antibodies. Quantification of (**A**) LAMP-1^+^ and (**B**) Ubiquitin^+^ DNs in 5xFAD;*Cx3cr1*^+*/*+^ (black bars) and 5xFAD;*Cx3cr1*^*−/−*^ (grey bars) mice. Error bars represent SEM. Statistical analysis done using two-tailed, standard Student t test with Welch’s correction for unequal SDs. ** *p* < 0.005 (**C**) nT-APP^+^ neurites (grey) associated with (solid arrows) compact vs. (dashed arrows) diffuse ThioS^+^ plaques (green) in the cortex of 4 month-old (top panels) 5xFAD;*Cx3cr1*^+*/*+^ and (bottom panels) 5xFAD;*Cx3cr1*^*−/−*^ mice. Scale bar = 30 µm. Quantification of (**D**) LAMP1^+^, (**E**) Ubiquitin1^+^ and (**F**) nT-APP^+^ DNs in the cortex of 5xFAD;*Cx3cr1*^+*/*+^ (black bars) and 5xFAD;*Cx3cr1*^*−/−*^ (grey bars) mice based on their size distribution at 4 months of age. < 500 µm = 50-500 µm, > 500 µm = 550-1000 µm. Data represents mean cortical DN abundance calculated using multiple sections from *n* = 6 mice (3 females, 3 males) of each genotype. Statistics done using two-way ANOVA (p.^int^ LAMP1, Ubiquitin-1 and nT-APP < 0.0001) with Tukey’s post-hoc tests.**p < 0.001, ***p < 0.0001, ****p < 0.00001, ns = not significant

**Fig. 6:**
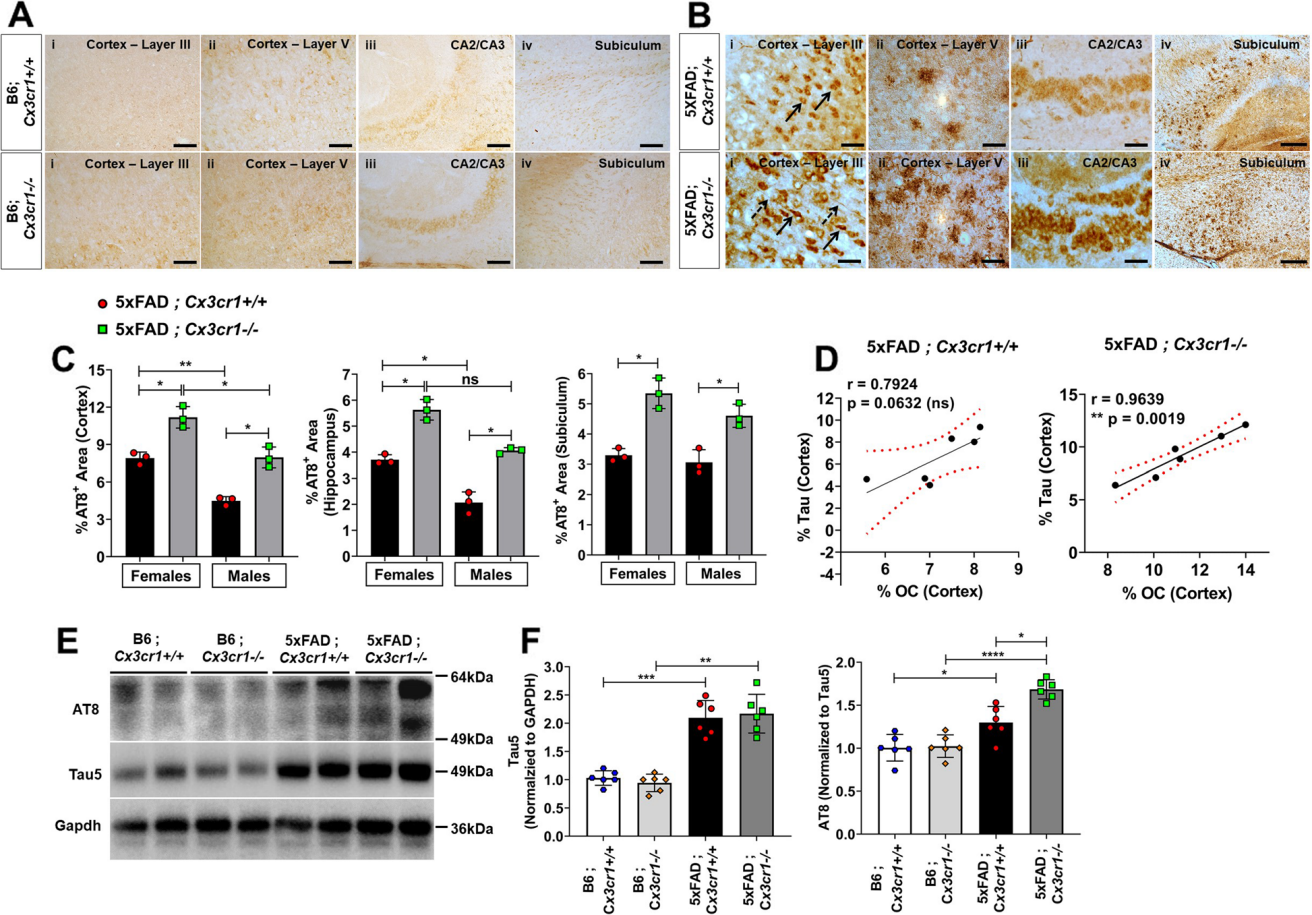
Aggravated *MAPT* pathology in 6 month-old 5xFAD;*Cx3cr1*^*−/−*^ mice. Distribution of AT8^+^ pTau in 6 month-old (**A**) B6;*Cx3cr1*^+*/*+^, B6;*Cx3cr1*^*−/−*^ and (**B**) 5xFAD;*Cx3cr1*^+*/*+^, 5xFAD;*Cx3cr1*^*−/−*^ mice. α-AT8 was used to label (6B-i) neuronal (solid arrows) and axonal (dashed arrows) pathology in cortical layer III, (6B-ii) dystrophic neurites in cortical layer V, (6B-iii) neuronal inclusions in CA2/3 and (6B-iv) neuritic plaques in the subiculum of 6 month old (top panels) 5xFAD;*Cx3cr1*^+*/*+^ and (bottom panels) 5xFAD;*Cx3cr1*^*−/−*^ mice. Images representative of pTau pathology in *n* = 6 mice (3 females, 3 males) in each genotype. Scale bars in A,B(i)-A(iii) = 50 µm. Scale bars in A,B(iv) = 100 µm. (**C**) Quantification of %AT8^+^ areas in the cortex, hippocampus, and subiculum of 6 month-old 5xFAD;*Cx3cr1*^+*/*+^ (black bars) and 5xFAD;*Cx3cr1*^*−/−*^ mice (grey bars). Data represents histological analyses performed using multiple, serial sections from *n* = 6 mice (3 females, 3 males), of each genotype. Error bars in C represent SEM. Statistical analysis for done using One-way Anova followed by Brown-Forsythe and Welch’s post-hoc tests. *p^adj^ < 0.01, **p^adj^ < 0.002. (**D**) Pearson’s Correlation analysis between %AT8^+^ Tau and %OC^+^ oAβ in cortices of 6 month-old 5xFAD;*Cx3cr1*^+*/*+^ and 5xFAD;*Cx3cr1*^*−/−*^ mice. (**E**) Western blot analyses to quantify the levels of soluble AT8^+^ pTau in the cortex of 6 month old 5xFAD;*Cx3cr1*^+*/*+^, 5xFAD;*Cx3cr1*^*−/−*^ and their genotype matched B6 controls. Blots representative of *n* = 6 (3 females, 3 males) per genotype. (**F**) Densitometric analysis to quantify fold changes in Tau5^+^ (total tau) and AT8^+^ (pTau) in the cortex of 6 month-old B6;*Cx3cr1*^+*/*+^ (white bars), B6;*Cx3cr1*^*−/−*^ (light grey bars), 5xFAD;*Cx3cr1*^+*/*+^ (black bars) and 5xFAD;*Cx3cr1*^*−/−*^ (dark grey bars) mice. Data for AT8 represents fold change in AT8 normalized to total tau levels. Error bars represent SEM. Statistical analysis for done using One-way Anova followed by Brown-Forsythe and Welch’s post-hoc tests. *p^adj^ < 0.01, **p^adj^ < 0.002, ***p^adj^ = 0.0002, ****p.^adj^ < 0.0001

**Fig. 7:**
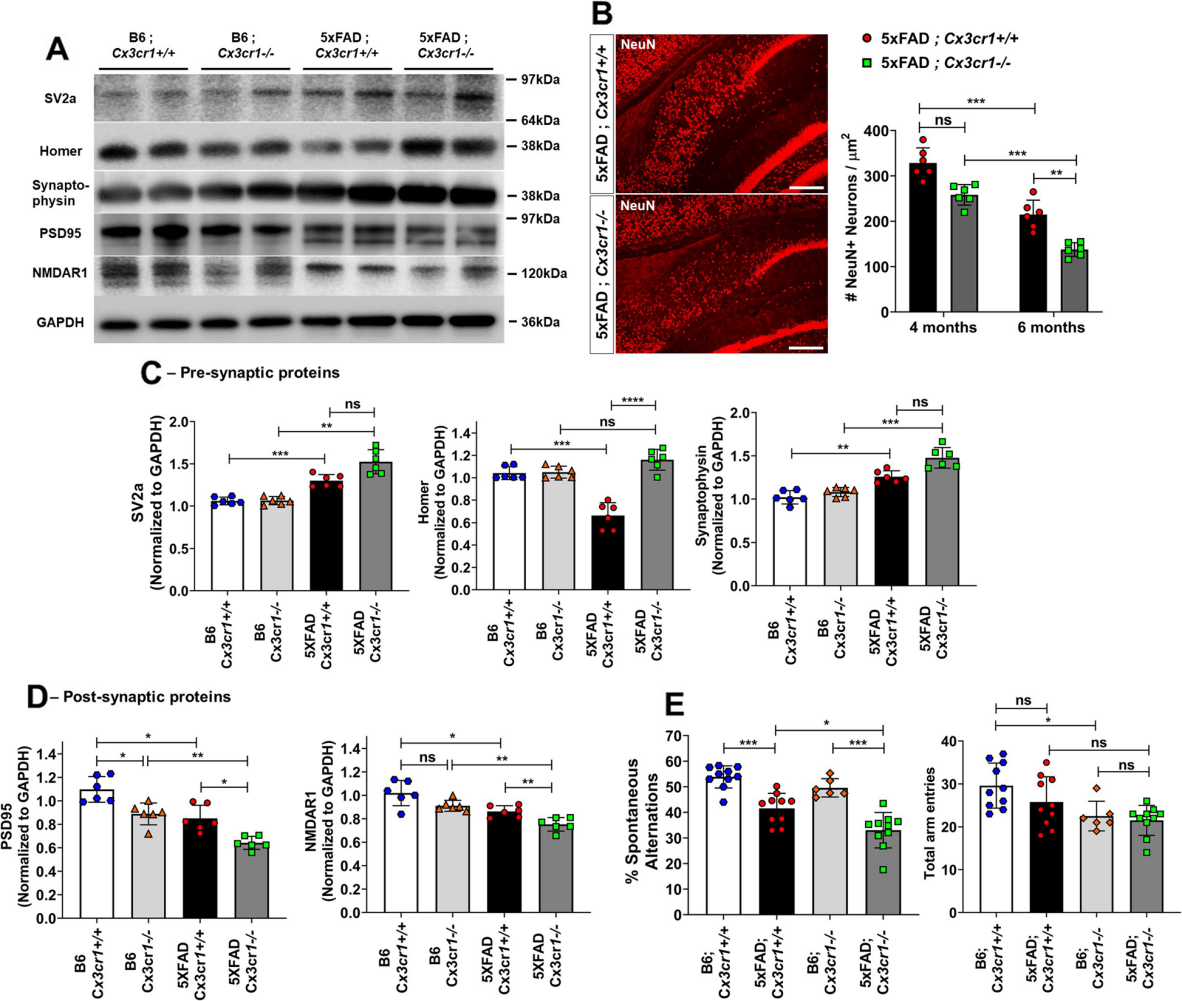
Aggravated synaptic dysfunction, neuronal loss and cognitive decline in the absence of *Cx3cr1*. (**A**) Representative western blots to assess the levels of pre-synaptic proteins—Synaptic Vesicle Protein-2 (SV2a), Synaptophysin and Homer, and post-synaptic proteins—Post-Synaptic Density-19 (PSD19) and NMDA Receptor subunit 1 (NR1) in cortical lysates of 6 month-old 5xFAD;*Cx3cr1*^+*/*+^ and 5xFAD;*Cx3cr1*^*−/−*^ mice and their genotype matched controls. Blots representative of *n* = 6 (3 females, 3 males) per genotype. (**B**) Visualization and quantitation of NeuN^+^ neuronal abundance in the subiculum of 4- and 6 month-old 5xFAD;*Cx3cr1*^+*/*+^ (black bars) and 5xFAD;*Cx3cr1*^*−/−*^ mice (grey bars). Images representative of NeuN^+^ neurons in subiculum of 6 month-old cohorts. Scale bars = 100 µm. Histological analyses and related quantification performed using multiple, serial sections from *n* = 6 mice (3 females, 3 males), of each genotype and at each time-point. Error bars represent SEM. Statistical analysis done using Two-way ANOVA (p^int^ = ns) followed by Tukey’s post-hoc tests. ***p = 0.0002, **p < 0.005. Densitometric analysis to quantify fold changes in (**C**) pre-synaptic proteins and (**D**) post-synaptic markers in cortical lysates of B6;*Cx3cr1*^+*/*+^ (white bars), B6;*Cx3cr1*^*−/−*^ (light grey bars), 5xFAD;*Cx3cr1*^+*/*+^ (black bars) and 5xFAD;*Cx3cr1*^*−/−*^ (dark grey bars) mice 5xFAD;*Cx3cr1*^*−/−*^ (dark grey bars). Fold changes were calculated using blots from *n* = 6 animals (3 females, 3 males) of each genotype run on the same gel to enable comparisons. Statistical analyses done using One-way ANOVA followed by Brown-Forsythe and Welch’s post-hoc tests. *p^adj^ < 0.05, **p^adj^ < 0.001, ***p^adj^ < 0.0001, ****p^adj^ < 0.00001. (**E**) Y-maze based evaluation of working memory in 6 month-old B6;*Cx3cr1*^+*/*+^ mice (white bars), B6;*Cx3cr1*^*−/−*^ (light grey bars), 5xFAD;*Cx3cr1*^+*/*+^ (black bars) and 5xFAD; *Cx3cr1*^*−/−*^ (dark grey bars). Behavioral testing done using ~ *n* = 10 animals (5 females, 5 males) per genotype. Statistical analysis done using One-way ANOVA followed by Brown-Forsythe and Welch’s post-hoc tests. *p^adj^ = 0.02, ***p.^adj^ < 0.0001, ns = not significant

## Results

### *Cx3cr1* deficiency does not alter the homoeostatic microglial phenotype in adult mice

Studies have shown a transient decrease in microglial densities in brains of B6;*Cx3cr1*^*−/−*^ mice between post-natal days 8 through 28 [12]. Similarly, sc-RNA seq studies that have demonstrated that transcriptomic differences observed in *FACS* purified, CD11b^+^CD45^+^ microglia from 2 month-old B6;*Cx3cr1*^*−/−*^ mice are not evident in aged microglia from 12 and 24 month-old *Cx3cr1* deficient mice as compared to cells isolated from age-matched B6;*Cx3cr1*^+*/*+^ animals [31]. These studies imply that *Cx3cr1* deficiency has transient and subtle effects on the transcriptional landscape of homeostatic microglia. To ascertain that no overt microglial defects persist into adulthood, we examined 6 month- old B6;*Cx3cr1*^+*/*+^ and B6;*Cx3cr1*^*−/−*^ mice for microglial abundance and homeostatic activation. qRT-PCR analyses using cortical mRNA revealed similar expression of canonical microglial genes namely *Pu.1*, *Iba1*, *P2ry12* and *Cd11b* (Supplementary Fig. 1Ai-Aiv). Furthermore, flow-cytometry analyses revealed similar numbers of CD11b^+^ cells in the brains of B6 mice with and without *Cx3cr1* (Supplementary Fig. 1B). Interestingly, while no differences were observed in the number of cellular processes between the two genotypes, *Cx3cr1*^*−/−*^ microglia displayed a modest increase in the number of process junctions as compared to microglia from B6;*Cx3cr1*^+*/*+^ mice (Supplementary Fig. 1C). Lastly, with the exception significantly reduced levels of *Cst7*, we observed no significant differences in the expression of genes associated with homeostatic or inflammatory microglia in 6 month-old B6;*Cx3cr1*^+*/*+^ and B6;*Cx3cr1*^*−/*−^ mice (Supplementary Fig. 4A, B). Taken together with these published studies, our data show similar homeostatic microglial signatures in 6 month-old B6 mice with and without *Cx3cr1*.

### *Cx3cr1* deficiency leads to accelerated plaque deposition

To investigate the kinetics of Aβ accumulation in the absence of *Cx3cr1*, brain sections from 4 and 6 month-old 5xFAD;*Cx3cr1*^+*/*+^ and 5xFAD;*Cx3cr1*^*−/−*^ mice were immunolabeled with anti-Aβ_1-42_ antibodies. As seen in our previous studies using APPPS1 mice [25, 26], loss of *Cx3cr1* resulted in significantly reduced MOAB2^+^ plaque load in the cortex and hippocampus in 4-month-old 5xFAD mice (Fig. 1A-C). By contrast, at 6 months of age, the number of MOAB2^+^ plaques were significantly increased in the cortex and hippocampus of 5xFAD;*Cx3cr1*^*−/−*^ mice as compared to age-matched 5xFAD;*Cx3cr1*^+*/*+^ animals (Fig. 1A-C). 6-month-old 5xFAD;*Cx3cr1*^+*/*+^ mice showed an ~ 1.5 fold and ~ twofold increase in Aβ_42_ plaque loads in the cortex and hippocampus respectively as compared to their 4 month-old counterparts. By contrast, an ~ 2.8 fold and ~ sixfold higher plaque burden in the cortex and hippocampus of 6 month of 5xFAD;*Cx3cr1*^*−/−*^ mice over 4 month-old 5xFAD;*Cx3cr1*^*−/−*^ animals indicated that plaque deposition is accelerated with disease progression in the absence of *Cx3cr1* (Fig. 1A-C).

### *Cx3cr1* deficiency exacerbates the accumulation of neurotoxic species of Aβ

Insoluble aggregates of fibrillar Aβ (fAβ) along with soluble, oligomeric Aβ (oAβ) are associated with neurotoxicity in AD [32–34]. To assess whether the loss of *Cx3cr1* alters the accumulation these neurotoxic Aβ species, we first stained serial brain sections with Thioflavin S (ThioS) to visualize fAβ plaques. ThioS^+^ plaques deposited in 5xFAD;*Cx3cr1*^*−/−*^ mice appeared significantly more diffuse when compared to those in 5xFAD;*Cx3cr1*^+*/*+^ mice (Fig. 1D). Using circularity analysis, to distinguish compact plaques from plaques with a filamentous/diffuse or an intermediate phenotype (Fig. 1E), we observed that *Cx3cr1* deficiency resulted in a significant reduction in the proportion of compact plaques in the cortex (Fig. 1F) and hippocampus (Fig. 1G) of 6-month-old 5xFAD mice, with a concomitant increase in accumulation of plaques with intermediate and diffuse morphologies. To investigate the accumulation of soluble oAβ species, we immune-stained for OC^+^ oAβ at 4- and 6- months of age. In-situ quantification of OC^+^ Aβ accumulation revealed that increased proportion of cortical areas were positive of OC immunoreactivity in 5xFAD;*Cx3cr1*^*−/*−^ mice throughout the course of the disease (Fig. 1H). Consistent with these results, high-resolution confocal microscopy revealed larger deposits of OC^+^ oAβ surrounding compact and filamentous ThioS^+^ plaques in the cortices of 6-month-old 5xFAD;*Cx3cr1*^*−/−*^ mice compared to similar plaques in 5xFAD;*Cx3cr1*^+*/*+^ mice (Fig. 1I). While female 5xFAD mice displayed significantly increased plaque burdens compared to males (Fig. 1B, C), no significant differences in OC^+^ oAβ loads were observed in female and male 5xFAD cohorts. Taken together, our results indicate that *Cx3cr1* deficiency shifts Aβ dynamics towards increased accumulation/generation of toxic species of soluble oAβ associated with highly filamentous fAβ plaques.

### Microglial engagement of ThioS.^+^ plaques is compromised in the absence of *Cx3cr1*

Effective microglial proliferation, followed by their recruitment to, and subsequent interaction with Aβ plaques has been associated with trimming of amyloid fibrils and reduced fibril-branching leading to plaque compaction. To investigate whether the shift towards accumulation of diffuse plaques in 5xFAD;*Cx3cr1*^*−/−*^ mice correlates with dysregulation of plaque associated microglial responses, we used flow-cytometry and histology to visualize microglia associated with fAβ plaques. Consistent with increased plaque burdens (Fig. 1), we observed increased numbers of CD11b^+^ microglia in the brains of 6 month-old 5xFAD;*Cx3cr1*^*−/−*^ mice (Supplemental Fig. 2Ai-Aiii), which corresponded to a significant increase in the proportion of cortical areas positive for Iba1 (%Iba1^+^,Supplemental Fig. 2Bi). Furthermore, when %Iba1^+^ areas were normalized to areas positive for ThioS^+^ fAβ, we observed no differences in the cortex of 6-month-old 5xFAD mice with or without *Cx3cr1* (Supplemental Fig. 2Bii). These data are reflective of efficient microgliosis in response to increased Aβ deposition in 5xFAD;*Cx3cr1*^*−/−*^ mice, and similar recruitment of Iba1^+^ microglia to fAβ plaques in 5xFAD animals regardless of *Cx3cr1* genotype. To further investigate *Cx3cr1*-dependent effects on proliferation of plaque-associated vs. non-plaque-associated microglia, we preformed stereological quantification of Ki67^+^ microglia in cortical layer V of 5xFAD;*Cx3cr1*^+*/*+^ and 5xFAD;*Cx3cr1*^*−/−*^ mice (Supplemental Fig. 2C-E). 6 month-old 5xFAD;*Cx3cr1*^*−/−*^ mice showed a modest but significant increase in the proportion of ThioS^+^ plaques associated with Ki67^+^ Pu.1^+^ Iba1^+^ microglia (Supplemental Fig. 2D). Additionally, we also observed a significant increase in the proportion of plaque associated Ki67^+^Iba1^+^ microglia in 5xFAD;*Cx3cr1*^*−/−*^ mice (Supplemental Fig. 2E). No non-plaque-associated Ki67^+^ Iba1^+^ cells were observed in 5xFAD mice regardless of their *Cx3cr1* genotype.

Interestingly, analysis of microglial plaque-engagement revealed that regardless of the plaque compaction phenotype, Iba1^+^ microglia formed well-defined barriers at the Aβ interface in 6 month-old 5xFAD;*Cx3cr1*^+*/*+^ mice (Fig. 2A). By contrast, Iba1^+^ plaque barriers appeared disorganized in 5xFAD;*Cx3cr1*^*−/−*^ cohorts (Fig. 2B). Next, we assessed whether *Cx3cr1* deficiency altered microglial plaque engagement throughout disease progression. 4 month-old 5xFAD;*Cx3cr1*^*−/−*^ mice showed significant reduction in Iba1^+^ process engagement of filamentous and intermediate ThioS^+^ plaques when compared to similar plaques in 5xFAD;*Cx3cr1*^+*/*+^ mice. No differences were observed in Iba1^+^ engagement of compact plaques in the presence or absence of *Cx3cr1* at this age (Fig. 2C). Interestingly, at 6 months, while diffuse ThioS^+^ plaques in 5xFAD;*Cx3cr1*^*−/−*^ mice displayed a similar reduction in Iba1^+^ process engagement, no differences were observed in the proportion of Iba1^+^ processes associated with intermediate plaques in 5xFAD;*Cx3cr1*^+*/*+^ vs. 5xFAD;*Cx3cr1*^*−/−*^ mice (Fig. 2D). By contrast, *Cx3cr1* deficiency significantly impaired microglial engagement of compact plaques at this age (Fig. 2D). Studies have shown that TREM2 levels are elevated in the AD brain, and TREM2 expression is enriched particularly in Iba1^+^ microglial processes that engage with Aβ plaques [28]. Microglial upregulation of TREM2 is not only critical for efficient plaque-engagement, but active TREM2-signaling at the microglia-Aβ interface is also critical for efficient plaque compaction [28]. qRT-PCR analyses of FACS purified, CD11b^+^ microglia from 6 month-old 5xFAD;*Cx3cr1*^+*/*+^ and 5xFAD;*Cx3cr1*^*−/−*^ mice revealed no differences in expression of microglial *Trem2* and its signaling partner, *Tyrobp* regardless of the *Cx3cr1* genotype (Supplemental Fig. 2F). Likewise, we found no differences in the concentration of total TREM2 in the cortical lysates of 6 month old 5xFAD;*Cx3cr1*^+*/*+^ and 5xFAD;*Cx3cr1*^*−/−*^ mice (Supplemental Fig. 2G). Taken together, these results indicate that CX3CR1-signaling shapes microglial plaque interaction without altering microglial proliferation and subsequent recruitment to Aβ deposits.

### 5xFAD;*Cx3cr1*.^*−/−*^ mice show altered apoptotic-signaling, ROS metabolism and oxidative stress responses

Given the aberrant accumulation of toxic Aβ and impaired microglial plaque engagement in 5xFAD;*Cx3cr1*^*−/−*^ mice, we hypothesized that the Aβ driven neuropathological milieu is altered in the absence of *Cx3cr1*. To investigate how CX3CR1 shapes glial activation and the neuroinflammatory microenvironment, we ran the nCounter® Neuroinflammation Panel which queries 770 genes involved in neuron-glia interactions, inflammation, and neuroplasticity (Supplemental Files 8, 9). Transcriptional analyses using cortical RNA from 6 month-old 5xFAD animals revealed upregulation of genes associated with cellular apoptosis (*Casp9, Casp3, Casp8*) and pro-survival signaling (*Bcl2l1*) in 5xFAD;*Cx3cr1*^*−/−*^ mice when compared to *Cx3cr1*^+*/*+^ counterparts (Fig. 3Ai-Aii). Interestingly, we observed increased expression of pro-inflammatory genes (*Ccl2*, *Ccl5*), along with increased *Cst7*, *P2ry17* and *Tgfbr1* levels in 5xFAD;*Cx3cr1*^*−/−*^ mice (Fig. 3Ai-Aii). Lastly, genes associated with nitric-oxide signaling, ROS production and oxidative stress responses were differentially altered in 5xFAD;*Cx3cr1*^*−/−*^ animals (*Pten, Pink1, Nostrin, Sod2, Anxa1, Lcn2*) (Fig. 3Ai-Aii). Gene-ontology analysis of differentially expressed genes (DEGs) revealed that in comparison with their female counterparts that displayed regulation of programmed cell death/apoptosis, regulation of ROS metabolism and regulation of TGFβ3 signaling as the key biological pathways affected by the loss of *Cx3cr1*, top processes affected in male 5xFAD;*Cx3cr1*^*−/−*^ mice were associated with signaling related to cell cycle arrest in response to DNA damage and ER stress (Supplemental Fig. 3A). Despite these differences, common pathways altered in female and male 5xFAD;*Cx3cr1*^*−/−*^ mice indicated increased cellular apoptosis/necroptosis, altered oxidative/ER stress, increased DNA damage and cell cycle arrest (Supplemental Fig. 3B). Additionally, KEGG enrichment analysis revealed that loss of CX3CR1 signaling may result in altered phagocytosis along with alterations in key signaling pathways with known involvement in intracellular protein shuttling, protein phosphorylation, cellular senescence, synaptic plasticity/transmission, and cognition (Supplemental Fig. 3C).

### *Cx3cr1 *deficiency drives dysregulated microglial activation in 5xFAD mice

To investigate whether the transcriptomic changes identified using the nCounter® system are specifically reflective of microglial dysregulation, we used FACS-purified CD11b^+^ microglia isolated from brains of 6 month-old 5xFAD;*Cx3cr1*^+*/*+^ and 5xFAD;*Cx3cr1*^*−/−*^ animals to validate top DEGs and biological pathways using qRT-PCR. Based on increased *P2ry12* expression in male 5xFAD;*Cx3cr1*^*−/−*^ mice (Fig. 3Aii), we first investigated the skewing of microglia towards a ‘disease-associated’ phenotype. While *Clec7a* and *Cst7* mRNA levels were upregulated in microglia from male and female 5xFAD;*Cx3cr1*^*−/−*^ mice (Fig. 3Bii, Biii), *Apoe* mRNA levels were elevated in microglia only in female 5xFAD;*Cx3cr1*^*−/−*^ mice, when compared to cells isolated from sex-matched 5xFAD;*Cx3cr1*^+*/*+^ mice (Fig. 3Bi). Interestingly, microglia from 5xFAD;*Cx3cr1*^*−/−*^ mice showed increased mRNA levels for *Tgfβ-r1* with reduced *Tgfβ1* expression (Fig. 3Biv, Bv), signaling components that have been implicated in a protective microglial phenotype [35]. Furthermore, microglia from 5xFAD;*Cx3cr1*^*−/−*^ brains express significantly increased mRNA levels of pro-inflammatory *Ccl2*, *Ccl5* and *Il-1β*, along with reduced *Tnf* and *Il-6* transcript levels (Fig. 3Ci – Cv). Taken together, these data indicate that *Cx3cr1* deficiency dysregulates microglial activation towards a phenotype primed for increased neurotoxicity. Lastly, increased mRNA levels of *Pten* (Fig. 3Di) and *Cybb/iNos* (Fig. 3Dii) coupled with reduction in *Pink1* (Fig. 3Diii) mRNA expression indicate that 5xFAD;*Cx3cr1*^*−/−*^ microglia display increased oxidative/mitochondrial stress responses. No significant differences in the expression of these genes in 6 month-old B6;*Cx3cr1*^+*/*+^ and B6;*Cx3cr1*^*−/−*^ mice (Supplemental Fig. 4) further confirmed that these differences in microglial activation elicited by *Cx3cr1* deficiency are indeed driven by AD pathology, and are not observed in the healthy, adult brain. Thus, the loss of CX3CR1-signaling exacerbates microglial dysfunction in AD, driving neurodegenerative activation.

### *Cx3cr1* deficiency impairs microglial Aβ phagocytosis and lysosomal activity

While our previous studies have shown that a deletion of *Cx3cr1* in B6 mice augments microglial uptake of exogenously injected Aβ_42_ [25], the role of CX3CR1 in microglial phagocytosis of endogenously produced, extracellular fAβ plaques in the context of accumulating AD pathology is largely unclear. Plaque accumulation in 5xFAD mice in the presence and absence of *Cx3cr1* (Fig. 1) suggests that while microglia may be efficient phagocytes in early disease, their Aβ uptake and clearance capacities are impaired with disease progression. Thus, to investigate whether the phagocytic and lysosomal dysfunction suggested by our transcriptomic data (Supplemental Fig. 3) was functionally evident in 5xFAD mice, we injected (i.p.) 4- and 6-month old 5xFAD;*Cx3cr1*^+*/*+^ and 5xFAD;*Cx3cr1*^*−/−*^ mice with methoxy-X04 and analyzed microglial uptake of endogenous fAβ using flow cytometry. Characterization of CD11b^+^ microglia based on their surface expression of CD45 (Supplemental Fig. 5A), revealed that while CD11b^+^ CD45^low^ microglia accounted for ~ 35% of fAβ^+^ phagocytic microglia (Supplemental Fig. 5C), ~ 70% of CD11b^+^ CD45^high^ microglia were fAβ^+^, indicating that CD11b^+^ CD45^high^ cells represent an activated, highly phagocytic microglial subpopulation in 5xFAD mice (Supplemental Fig. 5C). The absence of any fAβ^+^ microglia in methoxy-X04 treated B6 mice (Supplemental Fig. 5B) indicated that the fAβ^+^ populations observed in 5xFAD cohorts were indeed microglia that had actively phagocytosed fAβ.

We observed a modest increase in the proportion of fAβ^+^ CD11b^+^ microglia in 4 month-old 5xFAD;*Cx3cr1*^*−/−*^ mice (~ 44%) as compared to age-matched 5xFAD;*Cx3cr1*^+*/*+^ cohorts (~ 36%), corresponding to a mild, but significant increase in the fAβ^+^ CD11b^+^ CD45^high^ microglial subset in these animals (Supplementary Fig. 6A), suggesting increased microglial uptake of fAβ in 4 month-old 5xFAD;*Cx3cr1*^*−/−*^ mice. Interestingly, these microglial subsets were further categorized into methoxy-X04^low^ / fAβ^low^ and methoxy-X04^high^ / fAβ^high^ subpopulations (Supplemental Fig. 5C). To assess whether these varied microglial populations differed in lysosomal activation indicative of their ability of clearing phagocytosed Aβ, we quantified their lysosomal activity using Lysotracker-DeepRed™ (-DR), which specifically labels all acidified, phagocytic compartments. Significantly higher lysotracker-DR mean fluorescence intensities (MFI) in the fAβ^high^ subpopulations within the CD45^low^ and CD45^high^ microglia, indicated increased endolytic activation in these subsets as compared to fAβ^low^ populations (Supplemental Fig. 6B, C). Interestingly, while lysotracker-DR MFI for the fAβ^high^ CD11b^+^ CD45^low^ microglia in 5xFAD;*Cx3cr1*^*−/−*^ mice was significantly higher than the corresponding subset from 5xFAD;*Cx3cr1*^+*/*+^ mice (Supplemental Fig. 6B), lysotracker-DR MFIs were significant reduced in all fAβ^+^ populations within CD11b^+^ CD45^high^ microglia in 5xFAD;*Cx3cr1*^*−/−*^ mice (Supplemental Fig. 6C). These data indicate that despite modest increases in their phagocytic potentials, microglia in 4 month-old 5xFAD;*Cx3cr1*^*−/−*^ mice show significant deficits in their overall endolytic activation.

In contrast to 4-month old cohorts, we observed that ~ 26% of CD11b^+^ microglia from 5xFAD;*Cx3cr1*^*−/−*^ mice had internalized fAβ, as compared to ~ 46% fAβ^+^ microglia in age-matched 5xFAD;*Cx3cr1*^+*/*+^ mice (Fig. 4A). Furthermore, significant reductions in the fAβ^+^ CD11b^+^ CD45^low^ and fAβ^+^ CD11b^+^ CD45^high^ microglia in 5xFAD;*Cx3cr1*^*−/−*^ mice indicated that *Cx3cr1* deficiency profoundly impaired microglial Aβ phagocytosis at this age (Fig. 4C). Higher lysotracker-DR MFI of fAβ^low^ and fAβ^high^ microglia within CD45^low^ and CD45^high^ populations in 5xFAD;*Cx3cr1*^+*/*+^ mice as compared to microglia from B6;*Cx3cr1*^+*/*+^ controls, indicated increased lysosomal activation of phagocytic microglia in 5xFAD mice (Fig. 4B). Interestingly, lysotracker-DR MFIs were significantly reduced in all microglial populations expect for fAβ^+^ CD11b^+^ CD45^high^ microglia in 5xFAD;*Cx3cr1*^*−/−*^ mice when compared to similar subsets in 5xFAD;*Cx3cr1*^+*/*+^ cohorts (Fig. 4B, D). Taken together, these results demonstrate that a) endolytic dysfunction in microglia in the absence of CX3CR1-signaling begins early in the course of AD and b) CX3CR1-driven effects on lysosomal activation may be crucial in shaping microglial uptake of Aβ as disease progresses.

### *Cx3cr1* deficiency leads to severe neuritic dystrophy

Based on studies that have demonstrated the association of OC^+^ oAβ with dystrophic neurites [36], we next assessed whether increased oAβ in 5xFAD;*Cx3cr1*^*−/−*^ mice (Fig. 1H-I) results in heightened neuritic dystrophy. Using α-LAMP1, α-nT-APP and α-Ubiquitin to visualize neuritic, ThioS^+^ plaques, we observed that 4 month-old 5xFAD;*Cx3cr1*^*−/−*^ showed significantly reduced numbers of dystrophic neurites in their cortices (Fig. 5A, B). However, 5xFAD;*Cx3cr1*^*−/−*^ animals displayed larger foci of severe neuritic dystrophy (Fig. 5C), associated with compact (solid arrows) as well as filamentous (dashed arrows) ThioS^+^ plaques, when compared to similar plaques in 5xFAD;*Cx3cr1*^+*/*+^ mice (Fig. 5C). Indeed, when dystrophic neurites were quantified based on size distribution, we observed that while a majority of LAMP1^+^ (Fig. 5D), Ubiquitin1^+^ (Fig. 5E) and nT-APP^+^ (Fig. 5F) neurites in the cortex of 5xFAD;*Cx3cr1*^+*/*+^ mice were < 500 µm, dystrophic neurites in 4 month-old 5xFAD;*Cx3cr1*^*−/−*^ mice were largely > 500 µm in size. This data demonstrates that 4 month-old 5xFAD;*Cx3cr1*^*−/−*^ mice display more severe neurodegenerative changes despite reduced plaque loads (Fig. 1) Similar accumulation of larger dystrophic neurites with severe neuritic pathology was also observed in the cortex of 6 month-old 5xFAD;*Cx3cr1*^*−/−*^ mice (Supplementary Fig. 7A – E). Lastly, severe neuritic dystrophy in 5xFAD;*Cx3cr1*^*−/−*^ mice at this age was strongly correlated with increased accumulation of filamentous fAβ plaques (Supplementary Table 2). These data indicate that the severity of neuritic dystrophy in the absence of *Cx3cr1* may be driven by the profile of toxic Aβ species rather than abundance of Aβ plaques.

### *Cx3cr1* deficiency aggravates tau pathology in 5xFAD mice

To identify mechanisms of overt neuronal and synaptic loss, we investigated the accumulation of pTau in 5xFAD mice. 6 month-old 5xFAD mice showed increased pTau pathology as compared to age-matched B6 controls (Fig. 6A, B). While intraneuronal AT8^+^ pTau was observed in cortical layer III (Fig. 6B-i, solid arrows, top panel) and the CA2/CA3 region (Fig. 6B-iii,top panel), AT8^+^ pTau also accumulated in dystrophic neurites in cortical layer V (Fig. 6B-ii, top panel), and in neuritic plaques in the subiculum (Fig. 6B-iv, top panel) in 5xFAD;*Cx3cr1*^*+/+*^ mice. Interestingly, in addition to intraneuronal AT8^+^ pTau in the CA2/CA3 in 5xFAD;*Cx3cr1*^*−/−*^ mice (Fig. 6B-iii,lower panel), mislocalized pTau also accumulated in axonal projections in their cortex (Fig.6A-i, dashed arrows, lower panel). Moreover, while the majority of AT8^+^ pTau accumulated as dystrophic neurites around senile, neuritic plaques, 5xFAD;*Cx3cr1*^*−/−*^mice displayed heighted pTau^+^neuritic dystrophy as compared to 5xFAD;*Cx3cr1*^*+/+*^mice in cortical layer V (Fig. 6B-ii, lower panel) and the subiculum (Fig. 6B-iv, lower panel). Indeed, 6 month-old 5xFAD;*Cx3cr1*^*−/−*^ mice showed a significant increase in the areas of the cortex, hippocampus and subiculum positive for AT8^+^pTau as compared to sex-matched 5xFAD;*Cx3cr1*^*+/+*^ animals (Fig. 6C), indicating aggravated deposition of pathological pTau. We observed an ~ 2-fold increase in the level of total, soluble tau in the cortex of 5xFAD;*Cx3cr1*^*+/+*^ and 5xFAD;*Cx3cr1*^*−/−*^ mice when compared to genotype-matched B6 controls (Fig. 6E, F). Furthermore, we observed a significant increase in the levels of soluble, AT8^+^ pTau in 5xFAD;*Cx3cr1*^*+/+*^ with respect to B6;*Cx3cr1*^*+/+*^ controls, which were further increased in 5xFAD;*Cx3cr1*^*−/−*^ mice (Fig. 6E, F). Interestingly, we found a significant correlation between cortical pTau accumulation and levels of OC^+^ oAβ (Fig. 1H, i) in 5xFAD;*Cx3cr1*^*−/−*^ but not 5xFAD;*Cx3cr1*^*+/+*^ mice (Fig. 6D), in line with studies that have implicated these soluble oAβ species in mislocalization and spread of pTau [37]. Lastly, cortical pTau significantly correlated with compact fAβ plaques in 5xFAD;*Cx3cr1*^*+/+*^ mice (Pearson’s *r* = 0.82, p*<0.05). In contrast, significant correlation was observed between cortical pTau and intermediate fAβ (Pearson’s *r* – 0.94, p****<0.00001) and diffuse fAβ (Pearson’s *r* = 0.82, p**<0.003) in 5xFAD;*Cx3cr1*^*−/−*^ animals.

### *Cx3cr1* deficiency aggravates synaptic dysfunction, neuronal loss and cognitive decline in 5XFAD mice

To characterize how increased toxic Aβ levels (Fig. 1), pTau accumulation (Fig. 6) and dysfunctional microglial activation (Fig. 3) affect neurotoxicity, we analyzed the levels of pre- and post-synaptic elements in 6 month-old cohorts. Interestingly, we observed a modest but significant increase in the expression of pre-synaptic proteins SV2a and Synaptophysin, with a significant reduction in Homer in 6 month-old 5xFAD;*Cx3cr1*^+/+^ mice when compared to B6;*Cx3cr1*^+/+^ controls (Fig. 7A, C). While loss of pre-synaptic Homer was not observed in age-matched 5xFAD;*Cx3cr1*^−/−^ mice, no significant differences were seen in Sv2a and Synaptophysin in the 5xFAD;*Cx3cr1*^−/−^ mice compared to 5xFAD;*Cx3cr1*^+/+^ cohorts (Fig. 7A,C). In contrast, 5xFAD;*Cx3cr1*^+/+^ mice showed an ~ 1.3 fold reduction in post-synaptic proteins, namely PSD95 and NMDAR1 when compared to B6:*Cx3cr1*^+/+^ controls. 5xFAD;*Cx3cr1*^*−/−*^ mice showed ~ 1.6 fold reduction in PSD95 and NMDAR1 as compared to B6; *Cx3cr1*^−/−^ mice, and an ~ 1.2–1.3 fold decrease as compared to their 5xFAD; *Cx3cr1*^*+/+*^ counterparts (Fig. 7A, D). Next, quantitation of NeuN^+^ neurons in the subiculum, the area of the most robust and chronic Aβ pathology in 5xFAD mice, revealed no differences in overall neuronal numbers in 4 month-old 5xFAD mice with and without *Cx3cr1* (Fig. 7B). However, we observed a significant reduction in NeuN^+^ neurons in the subiculum of 5xFAD;*Cx3cr1*^*−/−*^ mice at 6 months when compared to age-matched 5xFAD;*Cx3cr1*^+*/*+^ mice (Fig. 7B), which correlated with increased pTau^+^ pathology in these areas (Pearson’s *r* = 0.92, p*** < 0.0001, Fig. 6B-iv, C), Taken together, our data indicates that *Cx3cr1* deficiency worsens neurodegeneration and synaptic dysfunction in 5xFAD mice. Finally, to assess whether the loss of *Cx3cr1* worsens cognitive decline, we measured spatial working memory in 6 month-old cohorts of B6 and 5xFAD mice with and without *Cx3cr1*. While there were no differences in total arm entries in 5xFAD;*Cx3cr1*^+/+^ and 5xFAD;*Cx3cr1*^−/−^ mice, we observed a significant reduction in the number of arm entries in B6;*Cx3cr1*^−/−^ mice as compared to B6;*Cx3cr1*^+/+^ controls (Fig. 7E). Significant reduction in spontaneous alternations between arms was observed in 5xFAD;*Cx3cr1*^+/+^ and 5xFAD;*Cx3cr1*^−/−^ mice when compared to genotype matched B6 controls (Fig. 7E). Moreover, significantly decreased spontaneous alternations in 5xFAD;*Cx3cr1*^−/−^ mice as compared to 5xFAD;*Cx3cr1*^+/+^ cohorts demonstrated that *Cx3cr1* deficiency aggravates cognitive impairment in AD.

## Discussion

Distinct microglial clusters associated with Aβ plaques, NFTs and dystrophic/degenerating neurons, which are further critically impacted by signaling pathways such as CX3CR1, TREM2, PLCγ2 etc. have been identified in the AD brain. These data indicate that the dynamic neurodegenerative micro-environment is defined not only by microglial interaction with specific pathological AD features, but also by interactions between multiple, microglia-specific signaling pathways [2–4]. While current literature in the field is largely focused on investigating microglial pathways that confer increased risk for developing AD (e.g. TREM2, APOE, PLCγ2), disease-modifying SNPs around microglial genes, particularly the loss-of-function CX3CR1-V249I variant, have been associated with increased neuronal loss and disease severity in macular degeneration, ALS and AD [7, 8, 38]. Taken together with sc-RNA seq studies identifying downregulation *Cx3cr1* in plaque associated microglia as a molecular event that shapes microglial activation in AD [20–22], CX3CR1-signaling has emerged as a critical determinant of long-term pathological outcomes in AD. However, the downstream effects of attenuated CX3CR1 on the microglial phenotype and subsequent mechanisms that impact neurodegenerative pathology in AD remain elusive, in part due to the lack of comprehensive, transgenic animal models [14, 39]. Using 5xFAD animals, we demonstrate that in addition to skewing the microenvironment towards the accumulation of increasingly neurotoxic Aβ species, the loss of CX3CR1 signaling in AD results in impaired microglial plaque engagement, dampened microglial Aβ phagocytosis along with impaired lysosomal activation and skewing of microglia towards a ‘degenerative’ phenotype. Accumulation of dysfunctional microglia is associated with aggravated neuritic dystrophy, synaptic dysfunction, tau hyperphosphorylation, increased neurodegeneration and cognitive impairment. Disease phenotypes observed in the absence of *Cx3cr1* are reminiscent of pathology seen in mouse models with AD associated SNPs in key microglial genes such as TREM2 and APOE [2, 21, 22, 28]. These data underscore that highly dynamic microglial responses in AD are simultaneously shaped by interactions between multiple signaling pathways. For instance, our data suggests elevations in microglial *Apoe* and *Il-1β* in 5xFAD;*Cx3cr1*^−/−^ mice (Fig. 3), which may drive neurotoxic microglial responses via TREM2-APOE signaling axis [21]. Studies using the PS19 mouse model of tauopathy have demonstrated that TREM2 signaling aggravates NFT pathology, associated with pro-inflammatory, *Il-1β*^+^ microglia [40]. Taken together with published studies showing that increased pTau pathology in hTau;*Cx3cr1*^−/−^ mice is driven by microglial IL-1β [23, 24], our data suggests that *Cx3cr1* deficiency may drive pTau accumulation in 5xFAD mice via microglial TREM2-IL1β signaling.

Neuronal CX3CL1, the ligand for microglial CX3CR1, is cleaved by ADAM10, ADAM17 and BACE1, resulting in a membrane-bound C-terminal fragment (-ct). CX3CL1-ct is in-turn cleaved by γ-secretase to release the intracellular domain of CX3CL1 (-ICD). Using transgenic 5xFAD mice that overexpress CX3CL1-ct (5xFAD;CX3CL1-ct), *Fan et. al.* have recently demonstrated that CX3CL1-ICD promotes neurogenesis, potentially via the TGFβ3-Smad2 pathway. 5xFAD;CX3CL1-ct mice show decreased plaque loads and reduced neuronal loss [27, 41]. Lastly, overexpression of CX3CL1 in the PS19 model of tauopathy, reduces synaptic and neuronal loss and improves memory and cognition [41]. Interestingly, TGFβ3-signaling, regulation of synaptic plasticity and learning/memory/cognition are among the top biological/cellular processes altered in 6-month-old 5xFAD;*Cx3cr1*^−/−^ mice (Supplemental Fig. 3). However, in contrast to results described above, we see a significant increase in neurotoxicity in the subiculum (Fig. 7), and increased accumulation of pTau^+^ neurons and dystrophic neurites in the subiculum, cortex and hippocampus (Fig. 6) in the absence of *Cx3cr1*. Thus, while CX3CL1 signaling to neurons and TGFβ3-mediated neurogenesis may potentially be upregulated in 5xFAD;*Cx3cr1*^*−/−*^ mice, our data suggest that this is not sufficient to reverse/reduce neuronal loss and ameliorate long-term cognitive decline. We hypothesize that synergistic effects of CX3CL1 signaling via microglial-CX3CR1 and CX3CL1 back-signaling to neurons are required for amelioration of pathology and increased neuronal preservation in AD.

Ultrastructural analyses of microglia in mice exposed to chronic social stress have revealed that *Cx3cr1* deficiency aggravates the accumulation of ‘dark microglia’. These cells display signs of oxidative stress, including an electron-dense cytoplasm and nucleoplasm, cytoplasmic fragmentation, dilation of Golgi- and ER membranes and mitochondrial alterations [42]. Pathological, *Cx3cr1*^−/−^ microglia show downregulation of homeostatic markers like P2RY12 and display extensive engulfment of presynaptic axon terminals and postsynaptic dendritic branches. Similar ‘dark’ microglia associated with Aβ plaques in APPPS1 mice [42–44] are postulated to be responsible for aberrant synaptic stripping and pathological remodeling of synaptic circuits. Interestingly, our transcriptomic analysis has identified dysregulation of NO/ROS metabolism, responses to oxidative and ER stress, synaptic plasticity and synaptic vesicle cycling in 6 month old 5xFAD;*Cx3cr1*^−/−^ mice (Supplemental Fig. 3B-C), along with increased mRNA expression for *Pten* and *Cybb/Nos2* with a decrease in *Pink1* levels in microglia purified from 5xFAD;*Cx3cr1*^−/−^ mice (Fig. 3). Furthermore, while we demonstrate a preferential loss of post-synaptic densities in 5xFAD;*Cx3cr1*^−/−^ mice (Fig. 7A, D), 5xFAD;*Cx3cr1*^+/+^ and 5xFAD;*Cx3cr1*^−/−^ animals display modest but significant increases in pre-synaptic Sv2a and Synaptophysin levels as compared to their genotype matched B6 controls (Fig. 7A, C). We hypothesize that a) the loss of *Cx3cr1* may result in a significant reduction in pre- and post-synaptic coupling into active synapses and b) an increased recruitment of signaling proteins to pre-synaptic terminals may be reflective of compensation for the lack of synaptic coupling/plasticity observed in our mice (Supplemental Fig. 3). Why post-synaptic terminals are particularly vulnerable in AD, and what drives aggravated post-synaptic loss in the absence of *Cx3cr1* is not completely understood and remains an important question for targeted neuroprotective therapies.

We show that the pathological microenvironment in 5xFAD;*Cx3cr1*^−/−^ mice comprises of increased levels of highly diffuse, fAβ plaques as well as toxic, soluble (OC^+^) oAβ (Fig. 1), suggesting an impairment in amyloid clearance mechanisms. Studies have indicated that prolonged exposure to toxic Aβ peptides downregulates expression of phagocytic receptors such as CD47, CD36 and RAGE [45], impairs microglial autophagy and induces lysosomal dysfunction in AD [46], an observation corroborated *in-vivo* by significant alteration of endocytosis/phagocytosis and AGE-RAGE signaling pathways (Supplemental Fig. 3) coupled with reduced fAβ uptake and lysosomal acidification in CD11b^+^ microglia from 5xFAD;*Cx3cr1*^*−/−*^ mice in this study (Supplemental Fig. 6, Fig. 4).

Studies using stereotaxic injections of pathological human-AD tau into 5xFAD and APP-KI mice have shown that seeding and spread of pTau is affected by plaque burdens, where increased Aβ loads exacerbate *MAPT* pathology and lead to worsening of memory deficits [47]. Interestingly, studies in the *ArcTau*, Tg2576 and hAPP-J20 mouse models of AD have shown that soluble, OC^+^ oAβ, rather than insoluble Aβ plaques facilitate pTau pathology and cognitive deficits [37, 48]. Recent GWAS studies that have implicated CX3CR1-V249I (rs3732349), a putative loss-of-function variant previously associated with age-related macular degeneration, in worsened NFT pathology and Braak staging in AD, and neurodegeneration in ALS [6–8, 38, 49]. In line with these data, we observe an overall increase in tau hyperphosphorylation and accumulation of AT8^+^ pTau in neuronal soma along with mislocalization of pTau into axons, that significantly correlates with OC^+^ oAβ accumulation in the absence of *Cx3cr1*. Furthermore, our previous work in hTau;*Cx3cr1*^−/−^ mice has implicated reactive, inflammatory, CD68^+^, IL1β^+^ microglia in active spread of neurotoxic tau [23, 24]. Our studies also show an increase in pro-inflammatory microglia in 5xFAD;*Cx3cr1*^−/−^ mice with increased transcript levels of *Ccl2*, *Ccl5* and *Il1β* along with a downregulation of *Tgfβ1* mRNA expression. Coupled with increased *Clec7a*, *Apoe* and *Cybb/Nos2* levels, microglia in 5xFAD;*Cx3cr1*^−/−^ mice show a phenotype reminiscent of ‘degenerative’ microglia, associated with apoptotic neurons in AD and EAE. This degenerative microglial phenotype correlates with an overall increase in neurodegenerative pathology including severe neuritic dystrophy, aggravated neuronal loss in the subiculum, and worsened memory deficits in 6 month-old 5xFAD;*Cx3cr1*^−/−^ mice. Interestingly, we observed increased levels of total tau in soluble fractions of 5xFAD brain lysates, as compared to their respective B6 controls. We hypothesize that this increase is reflective of the release of tau from degenerating / dying neurons into the interstitial space, which in-turn potentiates the seeding and spreading of neurotoxic tau [50]. We postulate that the increased availability of these soluble tau seeds, in conjunction with elevated oAβ and filamentous fAβ levels (Fig. 1, Fig. 6D) drive exacerbated pTau pathology observed in 5xFAD;*Cx3cr1*^−/−^ mice. Interestingly, *Fuhrmann et. al.* have demonstrated that the loss of *Cx3cr1 reduces* neuronal loss in cortical layer III in the 5xTg mouse model of AD [51]. This study showed that active polarization of microglial processes towards layer III neurons is required for neuronal elimination in 5xTg mice. Unlike our results which show heightened neuronal pTau aggregation in layer III (Fig. 6), eliminated neurons in 5xTg mice display increased aggerates of Aβ_42_ but not pTau. While we have not pursued an in-depth stereological quantification of cortical neurons, our preliminary observations indicate heightened neurodegeneration in cortices of 5xFAD;*Cx3cr1*^*−/−*^ mice.

Taken as a whole, these data indicate that a) active elimination of neurons by neurodegenerative microglia may be dictated by the quality of intraneuronal pathological protein aggregates and b) microglial neurodegenerative responses modulated by CX3CR1 may be subject to region-dependent microglial heterogeneity along with regional differences in neuronal susceptibility [44, 52, 53]. We hypothesize that aggravated pTau pathology and subsequent neuronal loss in 5xFAD;*Cx3cr1*^*−/−*^ mice may be potentiated, in part, by elevated levels of neurotoxic oAβ along increasingly neurotoxic, filamentous plaques, resulting in aggravated spread of neurotoxic tau by pathological, inflammatory microglia.

## Conclusions

Clinical outcomes in AD are a net sum of a range of pathological microglial activation states that are dynamically controlled by CX3CR1-signaling in response to the diverse pathological features of AD. Thus, CX3CR1 may simultaneously mediate distinct microglial phenotypes based on contextual cues received via their interaction with Aβ, dysfunctional synapses, dystrophic neurites or distressed neurons within their microenvironment. While our understanding of the overarching role of microglial CX3CR1 on disease mechanisms that affect neuronal and synaptic health is still primitive, this study is the first to provide an *in-vivo* link between CX3CR1-dependent microglial activation, aberrant homeostasis of soluble and insoluble Aβ and subsequent effects on chronic neurodegeneration in AD. This work suggests that the long term neurodegenerative changes including accumulation of pathological tau, synaptic dysregulation and altered neuronal homeostasis in the absence of *Cx3cr1* correlate with the *quality* along with the abundance of extracellular Aβ. We hypothesize that *Cx3cr1* deficiency in AD impairs microglial endolytic activation, thereby affecting uptake and degradation of fibrillar Aβ, and triggering an accumulation of neurotoxic Aβ species. Lastly, the neurotoxic Aβ microenvironment in the absence of *Cx3cr1* further drives increased microglial dysfunction typified by aberrant inflammatory activation, ROS metabolism and a skewing of the microglial response to a neurodegenerative phenotype.

## Supplementary Information

**Additional file 1: Supplemental Fig. 1. ***Cx3cr1* deficiency does not alter microglia in 6 month-old B6 mice. (**A**) Quantitative real time PCR (qRT-PCR) based quantification for cortical expression of microglial genes (i) *Pu.1*, (ii) *Iba1*, (iii) *P2ry12* and (iv) *Cd11b* in B6;*Cx3cr1*^*+/+*^ (black bars) and B6;*Cx3cr1*^*-/-*^ (grey bars) mice. Data represents mean ddCT values for *n* = 8 (4 females, 4 males) mice for each genotype. (**B**) Flow cytometry based quantitation of CD11b^+^ microglia in 6 month-old B6;*Cx3cr1*^*+/+*^ (black bars) and B6;*Cx3cr1*^*-/-*^ (grey bars) mice. Flow-cytometry plots representative of *n* = 8 mice (4 females, 4 males) for each genotype. (**C**) Representative, high-resolution confocal microscopy images of Iba1^+^ microglia from 6 month-old B6;*Cx3cr1*^*+/+*^ and B6;*Cx3cr1*^*-/-*^ mice used to quantify number of branches / cell, and junctions / cell. Branching and junctions quantified for a total of 72 microglia selected from *n* = 6 (3 females, 3 males, 12 microglia per animal) for each genotype. Statistical analysis done using two-tailed, standard Student’s t-test with Welch’s corrections for unequal SDs. *p = 0.02.

**Additional file 2: Supplemental Fig. 2.** *Cx3cr1* deficiency does not impair plaque associated microglial proliferation and microglial recruitment to Aβ plaques. (**A-i**) Representative flow-cytometry plots showing increased (**A-ii**) proportion and (**A-iii**) numbers of CD11b^+^ microglia in 6 month-old 5xFAD;*Cx3cr1*^+/+^ (black bars) and 5xFAD;*Cx3cr1*^-/-^ (grey bars) mice. Data represents mean microglial proportions / numbers quantified using *n* =6 (3 females, 3 males) mice for each genotype. Error bars represent SEM. Quantification of (**B-i**) %Iba1^+^ areas and (**B-ii**) %Iba1^+^ areas normalized to %ThioS^+^ areas in the cortices of 6 month-old 5xFAD;*Cx3cr1*^+/+^ (black bars) and 5xFAD;*Cx3cr1*^-/-^ (grey bars) mice. Error bars represent SEM. (**C**) Representative images of Ki67^+^Iba1^+^DapI^+^ proliferating microglia associated with diffuse ThioS^+^ plaques in 5xFAD;*Cx3cr1*^+/+^ (top panels) and 5xFAD;*Cx3cr1*^-/-^ (bottom panels) mice. Scale bars = 10µm. (**D**) Quantification of the proportion of ThioS^+^ plaques associated with proliferating microglia based on colocalization of Ki67, Pu.1 and Iba1 in cortices of 5xFAD;*Cx3cr1*^+/+^ (black bar) and 5xFAD;*Cx3cr1*^-/-^ (grey bar). (**E**) Quantification of proportions of Ki67^+^Iba1^+^DapI^+^ microglia associated with cortical, ThioS^+^ plaques in 6 month-old 5xFAD;*Cx3cr1*^+/+^ (black bars) and 5xFAD;*Cx3cr1*^-/-^ (grey bars) mice. Data in C-E processed using *n* = 6 (3 females, 3 males) of each genotype. Data in D, E based on analysis of 10,000 – 15,000 individual microglia associated with 250-300 plaques per genotype. (**F**) Quantitative real time PCR (qRT-PCR) analyses of *Trem2* and *Tyrobp* expression in CD11b^+^ microglia purified from *n* = 8 (females and males) 5xFAD;*Cx3cr1*^+/+^ and 5xFAD;*Cx3cr1*^-/-^ mice. (**G**) Quantitation of TREM2 protein levels in cortical lysates of 6 month-old 5xFAD;*Cx3cr1*^+/+^ (black bar) and 5xFAD;*Cx3cr1*^-/-^ (grey bar) mice. Lysates from age-matched 5xFAD;*Trem2*^-/-^ mice were used as negative controls. Data represents mean TREM2 concentrations in lysates from 6 mice (3 females, 3 males) of each genotype. Error bars represent SEM. Statistics in A, B and C calculated using two-tailed, standard Student’s t-test with Welch’s correction for unequal SDs. **p < 0.005, ***p = 0.002.

**Additional file 3: Supplemental Fig. 3.** Top biological processes and signaling pathways altered in 6 month-old 5xFAD;*Cx3cr1*^-/-^ mice when compared to 5xFAD;*Cx3cr1*^*+/+*^ mice. RNA extracts from cortical lysates from *n* = 6 animals (3 females, 3 males) were used for Nanostring based transcriptional profiling. (**A**) Gene ontology (GO) analyses showing the top 11 altered biological pathways in female vs. male 5xFAD;*Cx3cr1*^-/-^ mice with respect to sex-matched 5xFAD;*Cx3cr1*^+/+^ mice. Venn diagram highlighting (**B**) common biological pathways using GO analysis and (**C**) common neuropathology signaling pathways and cellular processes using KEGG analysis in male and female 5xFAD;*Cx3cr1*^-/-^ mice as compared to 5xFAD;*Cx3cr1*^+/+^ mice. GO and KEGG analysis done on significant differentially expressed genes (DEGs), which were calculated using the Benjamini-Hochberg test in the Nanostring nCounter Analysis Software.

**Additional file 4: Supplemental Fig. 4.** Microglial signatures, inflammatory activation and oxidative signaling are not altered by *Cx3cr1* deficiency in 6 month-old B6 mice. cDNA synthesized using cortical RNA from 6 month-old B6;*Cx3cr1*^*+/+*^ (black bars) and B6;*Cx3cr1*^*-/-*^ (grey bars) mice was used to perform qRT-PCR analyses. (**A**) Quantification of microglial activation phenotype assessed by gene-expression for (i) *Trem2*, (ii) *Clec7a*, (iii) *Cst7*, (iv) *Apoe*, (v) *Tgfβ1*, (vi) *Tgfβr1*, (vii) *Axl* and (viii) *Mertk*. (**B**) Inflammatory activation assessed by gene expression for (i) *Ccl2*, (ii) *Ccl5*, (iii) *Il-1β*, (iv) *Il-6* and (v) *Tnf*. (**C**) Quantification of oxidative stress signaling assessed by gene expression for (i) *Pten*, (ii) *Cybb*, (iii) *Pink-1*. Data represents mean ddCT values for *n* = 8 mice (4 females, 4 males) for each genotype. All data normalized to B6;*Cx3cr1*^*+/+*^ mice. Statistical analysis done using two-tailed, standard Student’s t-test with Welch’s corrections for unequal SDs. **p = 0.003.

**Additional file 5: Supplemental Fig. 5.** Flow cytometry based analysis of ex-vivo, microglial phagocytosis of fibrillar Aβ (fAβ). (**A**) Gating strategy to identify CD11b^+ ^CD45^low^ vs. CD11b^+ ^CD45^high^ microglia in methoxy-X04 injected 6 month-old cohorts. Flow cytometry plots shown represent data from B6;*Cx3cr1*^*+/+*^ and 5xFAD;*Cx3cr1*^*+/+*^ mice. (**B**) Flow cytometry plots showing the absence of non-specific retention of methoxy-X04 in CD11b^+^ microglia in B6;*Cx3cr1*^*+/+*^ mice and GFP^+^ microglia in B6;*Cx3cr1*^-/-^ mice. (**C**) Flow cytometry data showing proportions of methoxy-X04^+^ microglia within CD11b^+ ^CD45^low^ and CD11b^+ ^CD45^high^ subpopulations in 6 month-old 5xFAD;*Cx3cr1*^*+/+*^ mice. Microglia identified using gating strategy showed in (A). fAβ^+^ microglia are further classified into methoxy-X04^low^ / fAβ^low^ and methoxy-X04^high^ / fAβ^high^ sub-populations. (**D**) Flow cytometry data showing identification of microglia in 5xFAD;*Cx3cr1*^-/-^ mice based on CD11b^+^ vs. GFP^+^ expression for analysis of proportion of phagocytic, methoxy-X04^+^ sub-populations. Data in B and D show that the use of CD11b vs. GFP does not alter the profiles of fAβ^+^ microglia, and there is no non-specific, spectral overlap between CD11b, GFP and methoxy-X04 channels. All data representative of flow-cytometry analyses done using *n* = 5 female and 5 male mice of each genotype and processed in a single experiment. All experiments done using appropriate single-colored compensation controls to eliminate spectral overlap.

**Additional file 6: Supplemental Fig. 6.** Microglial fAβ uptake and endolytic activation is altered in 4 month-old 5xFAD;*Cx3cr1*^*-/-*^ mice. (**A**) Representative flow cytometry data showing methoxy-X04^+^/ fAβ^+^ CD11b^+^ microglia, and quantification of %Methoxy-X04^+^ cells within CD11b^+^ CD45^low^ and CD11b^+^ CD45^high^ microglial sub-populations in 5xFAD;*Cx3cr1*^*+/+*^ (black bars) and 5xFAD;*Cx3cr1*^*-/-*^ (grey bars) mice. Quantification of mean fluorescence intensities (MFI) of Lysotracker-DR for Methoxy-X04^low^ and Methoxy-X04^high^ microglia within (**B**) CD11b^+^ CD45^low^ and (**C**) CD11b^+^ CD45^high^ sub-populations in 5xFAD;*Cx3cr1*^*+/+*^ (black bars) and 5xFAD; *Cx3cr1*^*-/-*^ (grey bars) mice. All data is analyzed using *n* = 4 females for each genotype. Statistical analysis done using Two-way ANOVA followed by Tukey’s post-hoc tests.

**Additional file 7: Supplemental Fig. 7.** Larger dystrophic neurites (DNs) in 6 month-old 5xFAD;*Cx3cr1*^*-/-*^ mice. Representative, high-resolution confocal images for (**A**) LAMP1^+^ DNs and (**B**) Ubiquitin^+^ DNs associated with ThioS^+^ plaques in 6-month old 5xFAD;*Cx3cr1*^*+/+*^ and 5xFAD;*Cx3cr1*^*-/-*^ mice. Quantification of DNs in 6 month-old 5xFAD;*Cx3cr1*^*+/+*^ (black bars) and 5xFAD;*Cx3cr1*^*-/-*^ (grey bars) using (**A**) α-LAMP1, (**B**) α-Ubiquitin and (**C**) α-nT-APP antibodies. Dystrophic neurites were quantified on the basis of their size, defined as small (<500µm; 50-500µm) vs. large (>500µm; 550-1000µm). Data in C-E represents mean cortical dystrophic neurite abundance calculated using multiple sections from *n* = 6 mice (3 females, 3 males). Statistical analysis done using Two way ANOVA (pint for LAMP1^+^ DNs, Ubiquitin^+^ DNs and nT-APP^+^ DNs <0.0001) followed by Tukey’s post-hoc tests. *p <0.05, **p < 0.005, ns – non-significant.

**Additional file 8: Supplemental Table 1.**

**Additional file 9: Supplemental Table 2.** Pearson’s correlation analyses between cortical pTau pathology, small vs. large dystrophic neurites and plaque diffusivity in 6 month-old 5xFAD; *Cx3cr1*^*+/+*^ and 5xFAD; *Cx3cr1*^*-/-*^ mice. Pearson’s correlation analyses were done using data from Supplemental Figure 7 (size distribution of DNs in cortex), Figure 1F (cortical proportions of compact, intermediate and diffuse plaques) and Figure 6C (%AT8^+^ cortical areas) to investigate interactions between small (<500µm) and large (>500µm) (**A**) Ubiquitin^+^, (**B**) nT-APP^+^ and (**C**) LAMP1^+^ dystrophic neurites and their relation to compact vs. intermediate vs. diffuse plaques and accumulation of AT8^+^ pTau in the cortices of 6 month-old 5xFAD; *Cx3cr1*^*+/+*^ and 5xFAD; *Cx3cr1*^*-/-*^ mice. All data calculated using *n* = 6 (3 females, 3 males) of each genotype as described in materials and methods and related figure legends. Correlation matrices were calculated using imageJ. All data was tested for normality using the Anderson-Darling Test, and the Sharpiro-Wilk test for normality distribution. Significances were calculated using standard two-tailed t-tests with a 95% confidence interval. All significant interactions have been indicated in red. *p = 0.01, **p < 0.001, ns = not significant.

**Additional file 10.** Supplemental Materials and methods.

## Data Availability

All data supporting the conclusions of this article are included within the article and in additional files provided.

## Abbreviations

AD: Alzheimer’s disease
ALS: Amyotrophic Lateral Sclerosis
Aβ: Amyloid-β
DAB: Diaminobenzidine
DEGs: Differentially expressed genes
DNA: Deoxyribonucleic acid.
DNs: Dystrophic Neurites
EAE: Experimental autoimmune encephalomyelitis
EGFP: Enhanced green fluorescent protein
ER: Endoplasmic reticulum
FACS: Fluorescence activated cell sorting
fAβ: Fibrillar Amyloid-β
GO: Gene Ontology
GWAS: Genome wide association studies
HRP: Horse radish peroxidase
i.p.: Intra peritoneal
KEGG: Kyoto Encyclopedia of Genes and Genomes
LAMP1: Lysosome associated membrane protein-1
Mab: Monoclonal antibody
MFI: Mean Fluorescence Intensity
NFTs: Neurofibrillary tangles
NMDAR1: N-methyl-D-aspartate receptor subunit 1
NO: Nitric Oxide
nT-APP: N-terminal Amyloid Precursor Protein
oAβ: Oligomeric Amyloid-β
PBS: Phosphate buffered saline
PE: Phycoerythrin
PeCy7: Phycoerythrin-cyanin7
PSD95: Post synaptic density-95
pTau: (Hyper)phosporylated tau
ROI: Region of interest
ROS: Reactive Oxygen Species
SNP: Single Nucleiotide polymorphism
SOD1: Superoxide dismutase-1
SV2: Synaptic Vessicle-2
TREM2: Triggering receptor expressed on myeloid cells 2
