# Supplementary figures and images for: CX3CR1 deficiency aggravates amyloid driven neuronal pathology and cognitive decline in Alzheimer’s disease
Source: Mol Neurodegener. 2022 Jun 28;17:47. doi: 10.1186/s13024-022-00545-9 (PMC9241248; doi:10.1186/s13024-022-00545-9)

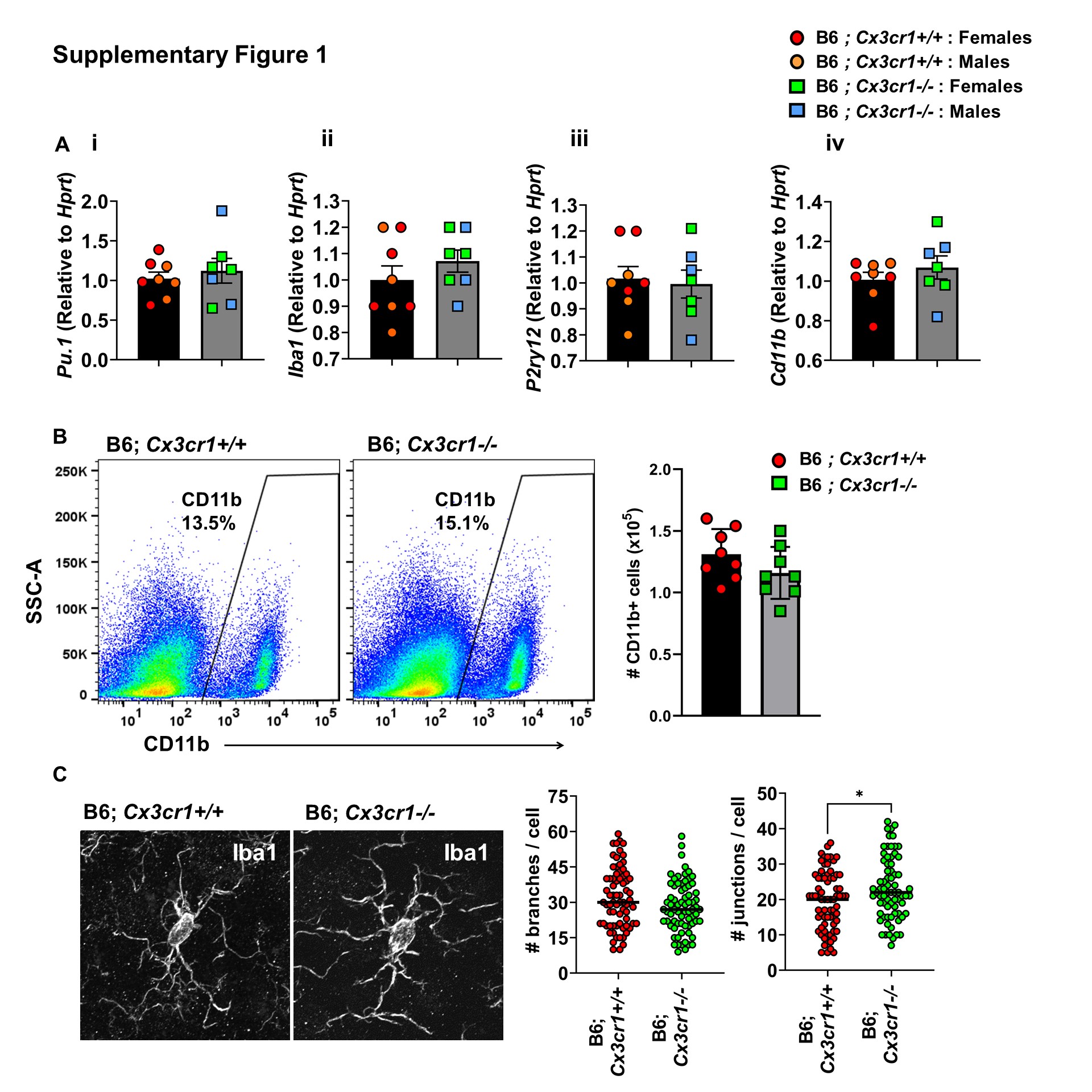

Supplement: Supplementary file 1 — Additional file 1: Supplemental Fig. 1. Cx3cr1 deficiency does not alter microglia in 6 month-old B6 mice. (A) Quantitative real time PCR (qRT-PCR) based quantification for cortical expression of microglial genes (i) Pu.1, (ii) Iba1, (iii) P2ry12 and (iv) Cd11b in B6;Cx3cr1+/+ (black bars) and B6;Cx3cr1-/- (grey bars) mice. Data represents mean ddCT values for n = 8 (4 females, 4 males) mice for each genotype. (B) Flow cytometry based quantitation of CD11b+ microglia in 6 month-old B6;Cx3cr1+/+ (black bars) and B6;Cx3cr1-/- (grey bars) mice. Flow-cytometry plots representative of n = 8 mice (4 females, 4 males) for each genotype. (C) Representative, high-resolution confocal microscopy images of Iba1+ microglia from 6 month-old B6;Cx3cr1+/+ and B6;Cx3cr1-/- mice used to quantify number of branches / cell, and junctions / cell. Branching and junctions quantified for a total of 72 microglia selected from n = 6 (3 females, 3 males, 12 microglia per animal) for each genotype. Statistical analysis done using two-tailed, standard Student’s t-test with Welch’s corrections for unequal SDs. *p = 0.02. [file 13024_2022_545_MOESM1_ESM.jpg]

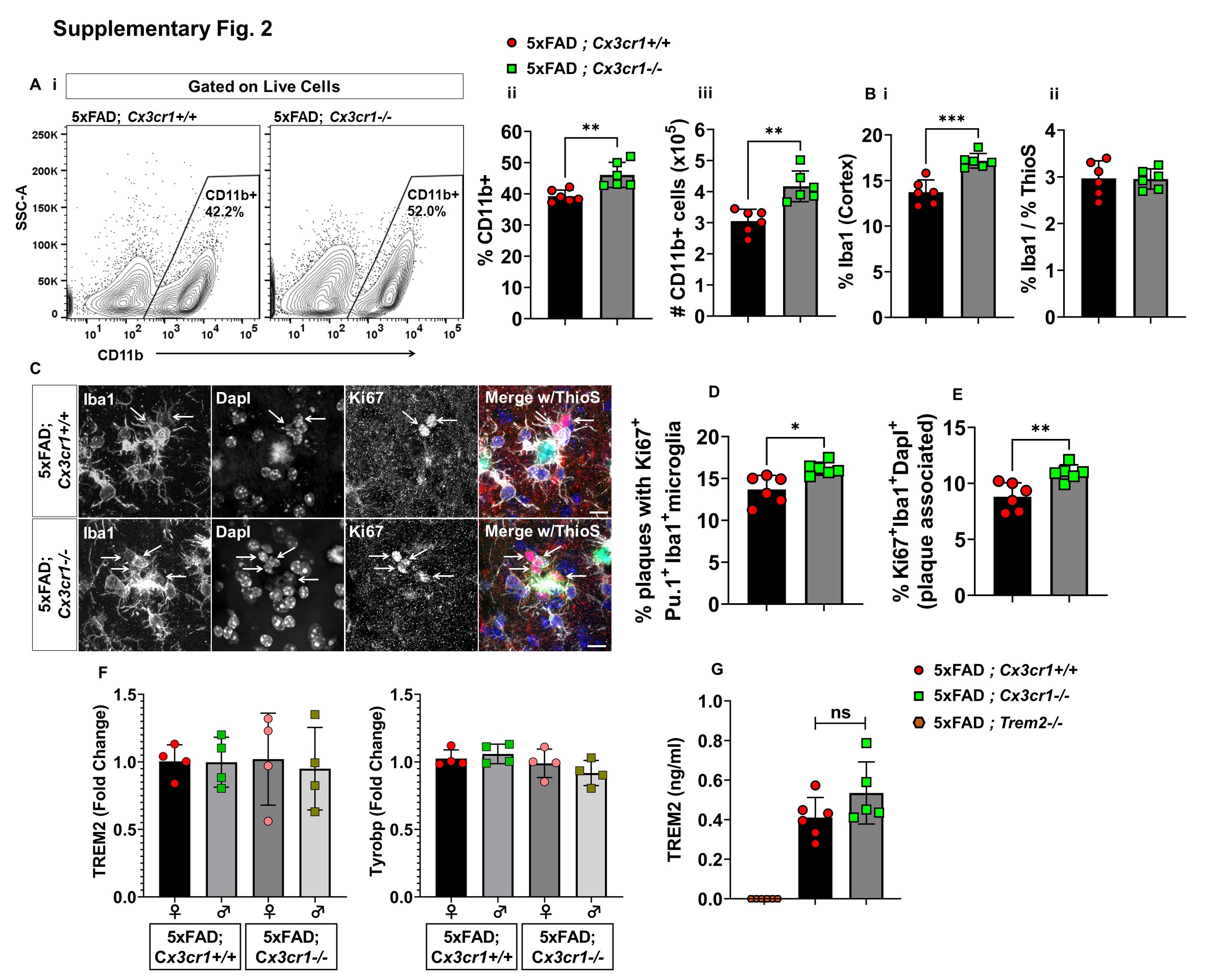

Supplement: Supplementary file 2 — Additional file 2: Supplemental Fig. 2. Cx3cr1 deficiency does not impair plaque associated microglial proliferation and microglial recruitment to Aβ plaques. (A-i) Representative flow-cytometry plots showing increased (A-ii) proportion and (A-iii) numbers of CD11b+ microglia in 6 month-old 5xFAD;Cx3cr1+/+ (black bars) and 5xFAD;Cx3cr1-/- (grey bars) mice. Data represents mean microglial proportions / numbers quantified using n =6 (3 females, 3 males) mice for each genotype. Error bars represent SEM. Quantification of (B-i) %Iba1+ areas and (B-ii) %Iba1+ areas normalized to %ThioS+ areas in the cortices of 6 month-old 5xFAD;Cx3cr1+/+ (black bars) and 5xFAD;Cx3cr1-/- (grey bars) mice. Error bars represent SEM. (C) Representative images of Ki67+Iba1+DapI+ proliferating microglia associated with diffuse ThioS+ plaques in 5xFAD;Cx3cr1+/+ (top panels) and 5xFAD;Cx3cr1-/- (bottom panels) mice. Scale bars = 10µm. (D) Quantification of the proportion of ThioS+ plaques associated with proliferating microglia based on colocalization of Ki67, Pu.1 and Iba1 in cortices of 5xFAD;Cx3cr1+/+ (black bar) and 5xFAD;Cx3cr1-/- (grey bar). (E) Quantification of proportions of Ki67+Iba1+DapI+ microglia associated with cortical, ThioS+ plaques in 6 month-old 5xFAD;Cx3cr1+/+ (black bars) and 5xFAD;Cx3cr1-/- (grey bars) mice. Data in C-E processed using n = 6 (3 females, 3 males) of each genotype. Data in D, E based on analysis of 10,000 – 15,000 individual microglia associated with 250-300 plaques per genotype. (F) Quantitative real time PCR (qRT-PCR) analyses of Trem2 and Tyrobp expression in CD11b+ microglia purified from n = 8 (females and males) 5xFAD;Cx3cr1+/+ and 5xFAD;Cx3cr1-/- mice. (G) Quantitation of TREM2 protein levels in cortical lysates of 6 month-old 5xFAD;Cx3cr1+/+ (black bar) and 5xFAD;Cx3cr1-/- (grey bar) mice. Lysates from age-matched 5xFAD;Trem2-/- mice were used as negative controls. Data represents mean TREM2 concentrations in lysates from 6 mice (3 females, 3 males) [file 13024_2022_545_MOESM2_ESM.jpg]

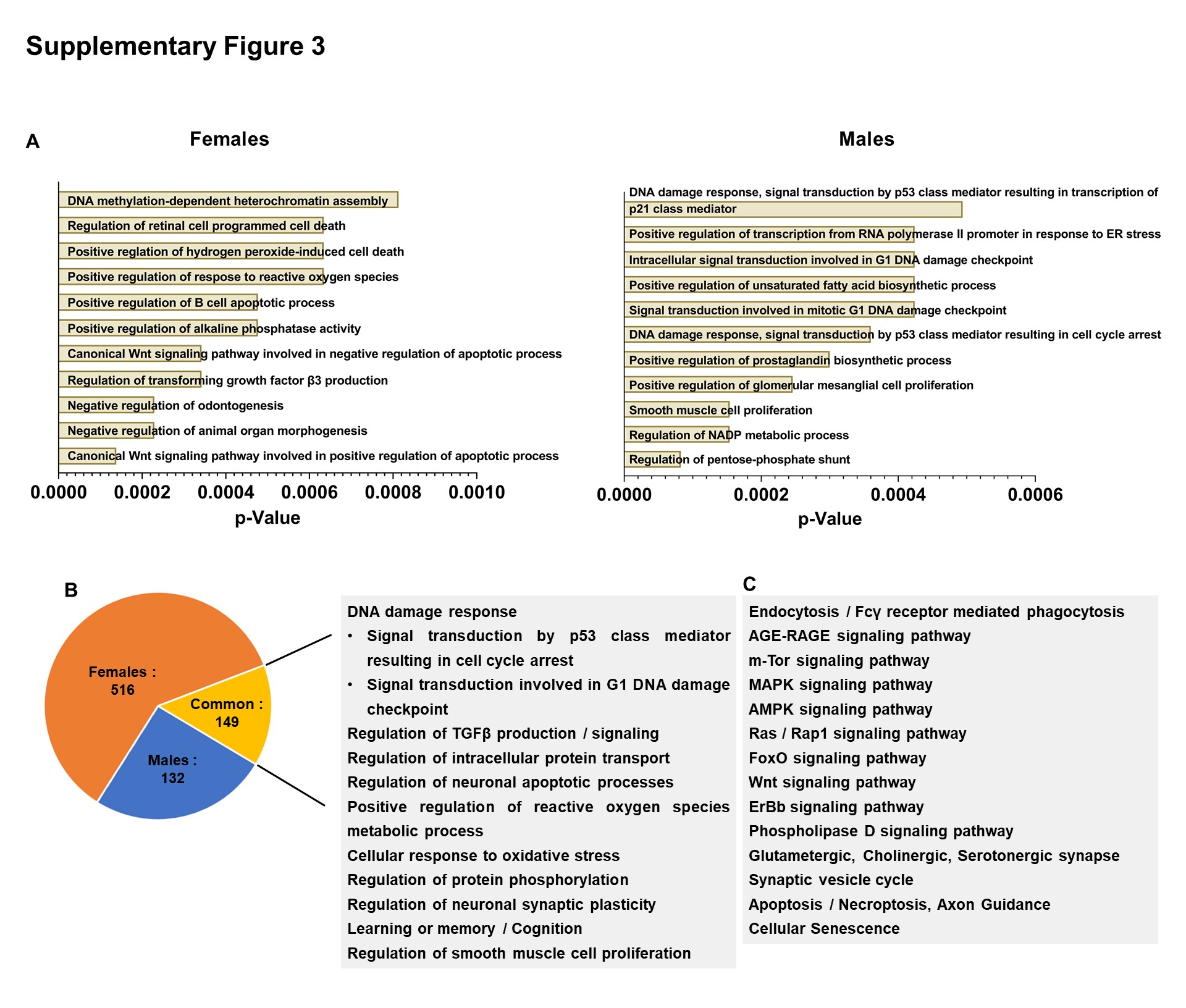

Supplement: Supplementary file 3 — Additional file 3: Supplemental Fig. 3. Top biological processes and signaling pathways altered in 6 month-old 5xFAD;Cx3cr1-/- mice when compared to 5xFAD;Cx3cr1+/+ mice. RNA extracts from cortical lysates from n = 6 animals (3 females, 3 males) were used for Nanostring based transcriptional profiling. (A) Gene ontology (GO) analyses showing the top 11 altered biological pathways in female vs. male 5xFAD;Cx3cr1-/- mice with respect to sex-matched 5xFAD;Cx3cr1+/+ mice. Venn diagram highlighting (B) common biological pathways using GO analysis and (C) common neuropathology signaling pathways and cellular processes using KEGG analysis in male and female 5xFAD;Cx3cr1-/- mice as compared to 5xFAD;Cx3cr1+/+ mice. GO and KEGG analysis done on significant differentially expressed genes (DEGs), which were calculated using the Benjamini-Hochberg test in the Nanostring nCounter Analysis Software. [file 13024_2022_545_MOESM3_ESM.jpg]

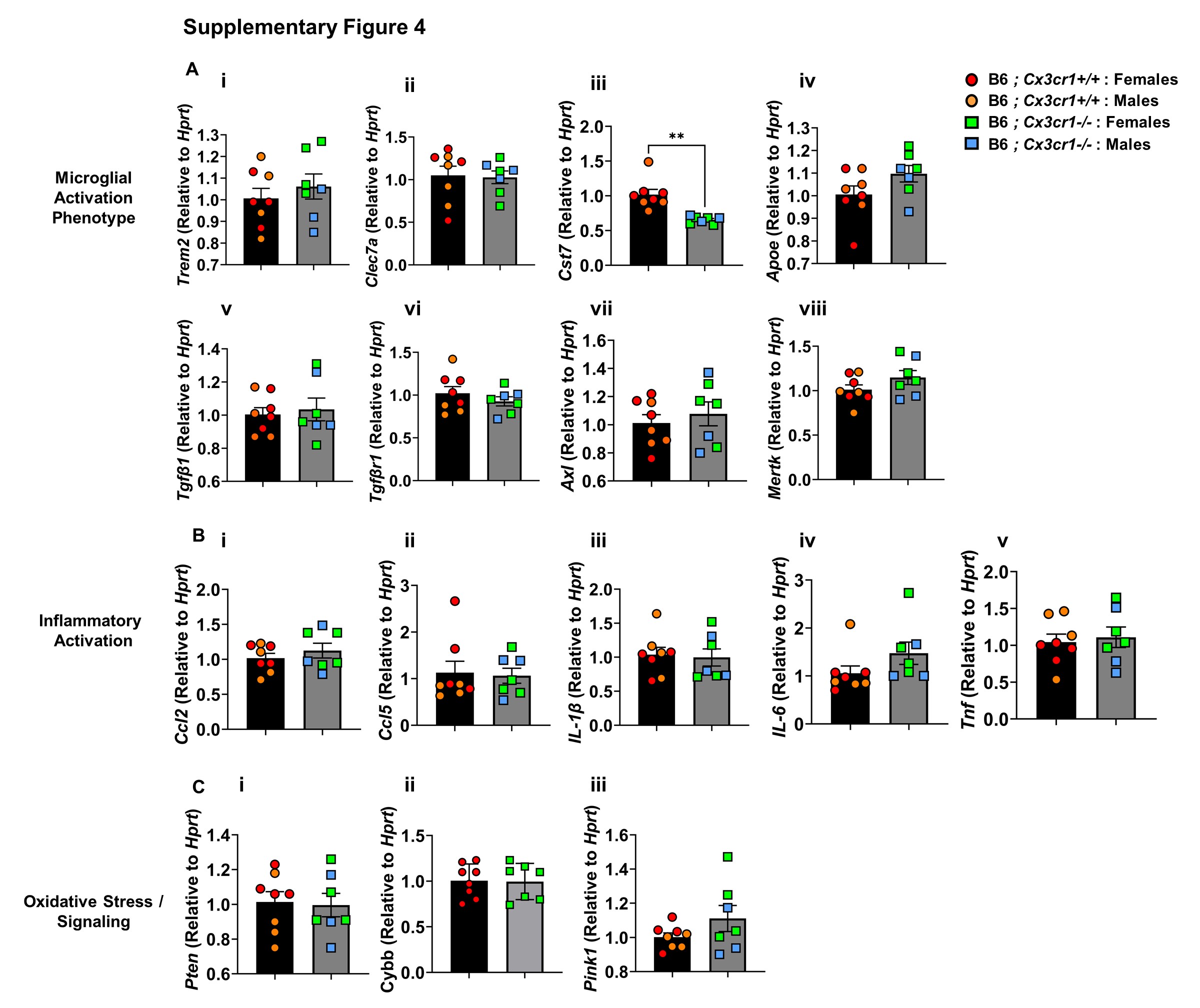

Supplement: Supplementary file 4 — Additional file 4: Supplemental Fig. 4. Microglial signatures, inflammatory activation and oxidative signaling are not altered by Cx3cr1 deficiency in 6 month-old B6 mice. cDNA synthesized using cortical RNA from 6 month-old B6;Cx3cr1+/+ (black bars) and B6;Cx3cr1-/- (grey bars) mice was used to perform qRT-PCR analyses. (A) Quantification of microglial activation phenotype assessed by gene-expression for (i) Trem2, (ii) Clec7a, (iii) Cst7, (iv) Apoe, (v) Tgfβ1, (vi) Tgfβr1, (vii) Axl and (viii) Mertk. (B) Inflammatory activation assessed by gene expression for (i) Ccl2, (ii) Ccl5, (iii) Il-1β, (iv) Il-6 and (v) Tnf. (C) Quantification of oxidative stress signaling assessed by gene expression for (i) Pten, (ii) Cybb, (iii) Pink-1. Data represents mean ddCT values for n = 8 mice (4 females, 4 males) for each genotype. All data normalized to B6;Cx3cr1+/+ mice. Statistical analysis done using two-tailed, standard Student’s t-test with Welch’s corrections for unequal SDs. **p = 0.003. [file 13024_2022_545_MOESM4_ESM.jpg]

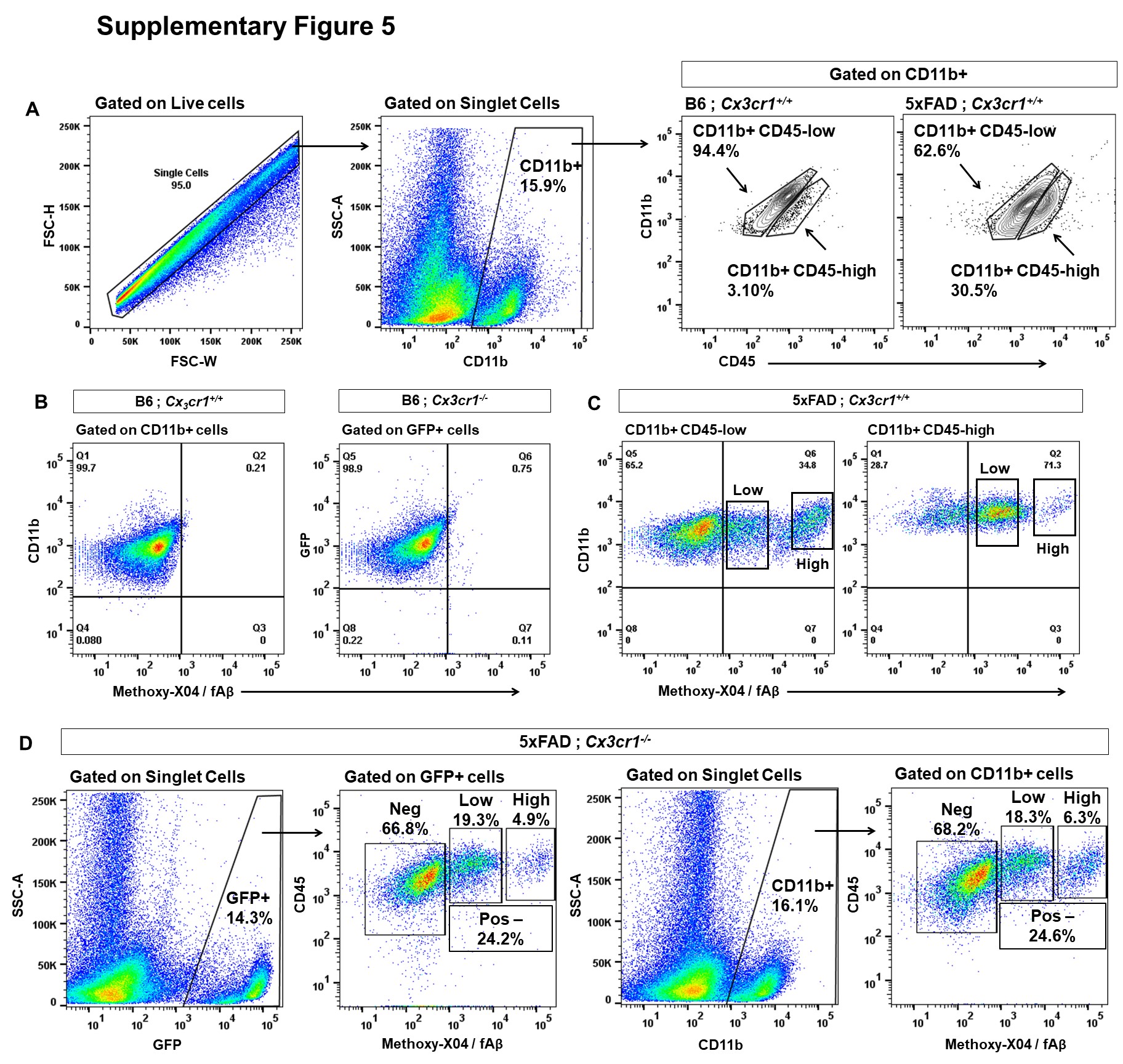

Supplement: Supplementary file 5 — Additional file 5: Supplemental Fig. 5. Flow cytometry based analysis of ex-vivo, microglial phagocytosis of fibrillar Aβ (fAβ). (A) Gating strategy to identify CD11b+ CD45low vs. CD11b+ CD45high microglia in methoxy-X04 injected 6 month-old cohorts. Flow cytometry plots shown represent data from B6;Cx3cr1+/+ and 5xFAD;Cx3cr1+/+ mice. (B) Flow cytometry plots showing the absence of non-specific retention of methoxy-X04 in CD11b+ microglia in B6;Cx3cr1+/+ mice and GFP+ microglia in B6;Cx3cr1-/- mice. (C) Flow cytometry data showing proportions of methoxy-X04+ microglia within CD11b+ CD45low and CD11b+ CD45high subpopulations in 6 month-old 5xFAD;Cx3cr1+/+ mice. Microglia identified using gating strategy showed in (A). fAβ+ microglia are further classified into methoxy-X04low / fAβlow and methoxy-X04high / fAβhigh sub-populations. (D) Flow cytometry data showing identification of microglia in 5xFAD;Cx3cr1-/- mice based on CD11b+ vs. GFP+ expression for analysis of proportion of phagocytic, methoxy-X04+ sub-populations. Data in B and D show that the use of CD11b vs. GFP does not alter the profiles of fAβ+ microglia, and there is no non-specific, spectral overlap between CD11b, GFP and methoxy-X04 channels. All data representative of flow-cytometry analyses done using n = 5 female and 5 male mice of each genotype and processed in a single experiment. All experiments done using appropriate single-colored compensation controls to eliminate spectral overlap. [file 13024_2022_545_MOESM5_ESM.jpg]

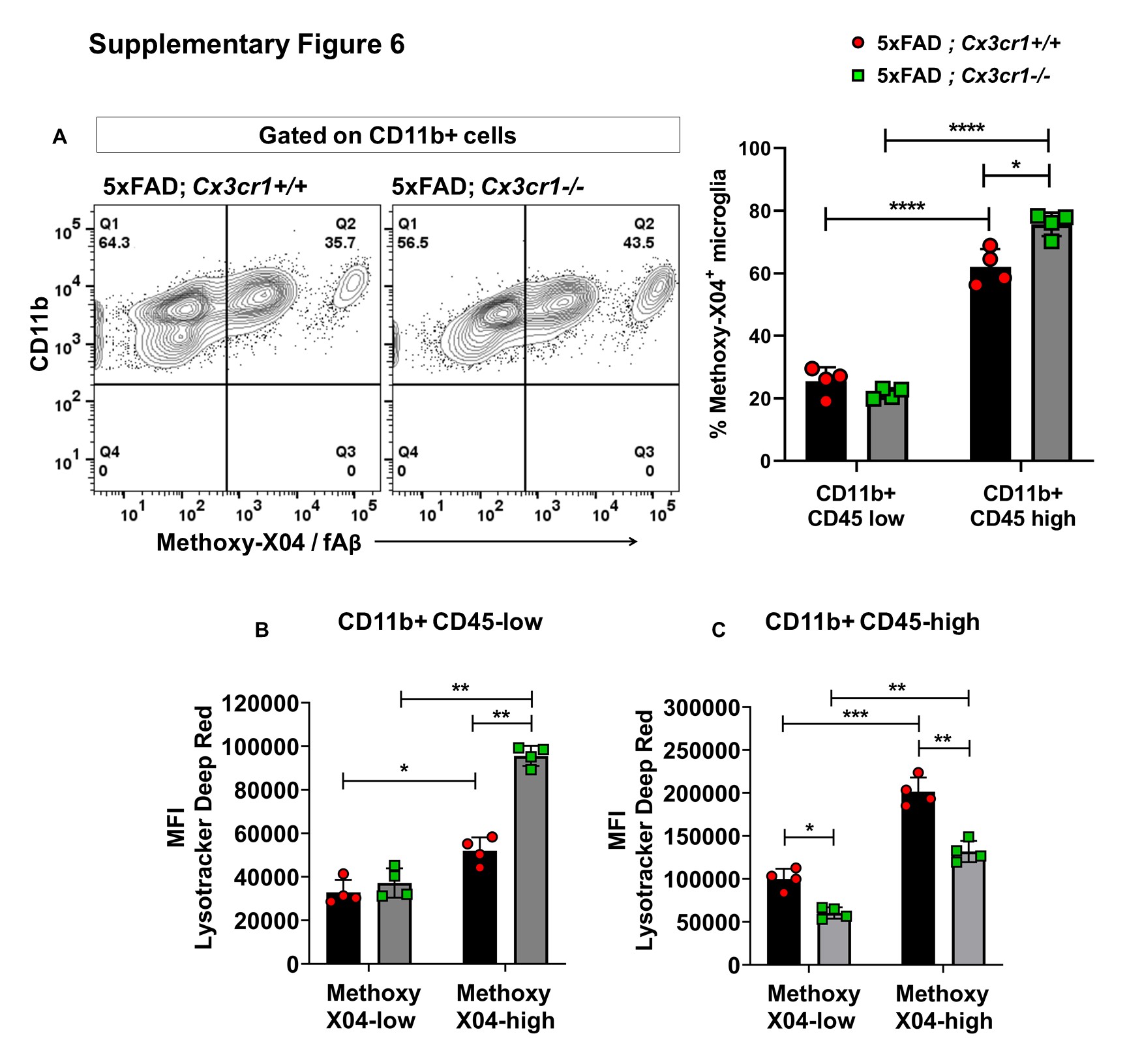

Supplement: Supplementary file 6 — Additional file 6: Supplemental Fig. 6. Microglial fAβ uptake and endolytic activation is altered in 4 month-old 5xFAD;Cx3cr1-/- mice. (A) Representative flow cytometry data showing methoxy-X04+/ fAβ+ CD11b+ microglia, and quantification of %Methoxy-X04+ cells within CD11b+ CD45low and CD11b+ CD45high microglial sub-populations in 5xFAD;Cx3cr1+/+ (black bars) and 5xFAD;Cx3cr1-/- (grey bars) mice. Quantification of mean fluorescence intensities (MFI) of Lysotracker-DR for Methoxy-X04low and Methoxy-X04high microglia within (B) CD11b+ CD45low and (C) CD11b+ CD45high sub-populations in 5xFAD;Cx3cr1+/+ (black bars) and 5xFAD; Cx3cr1-/- (grey bars) mice. All data is analyzed using n = 4 females for each genotype. Statistical analysis done using Two-way ANOVA followed by Tukey’s post-hoc tests. [file 13024_2022_545_MOESM6_ESM.jpg]

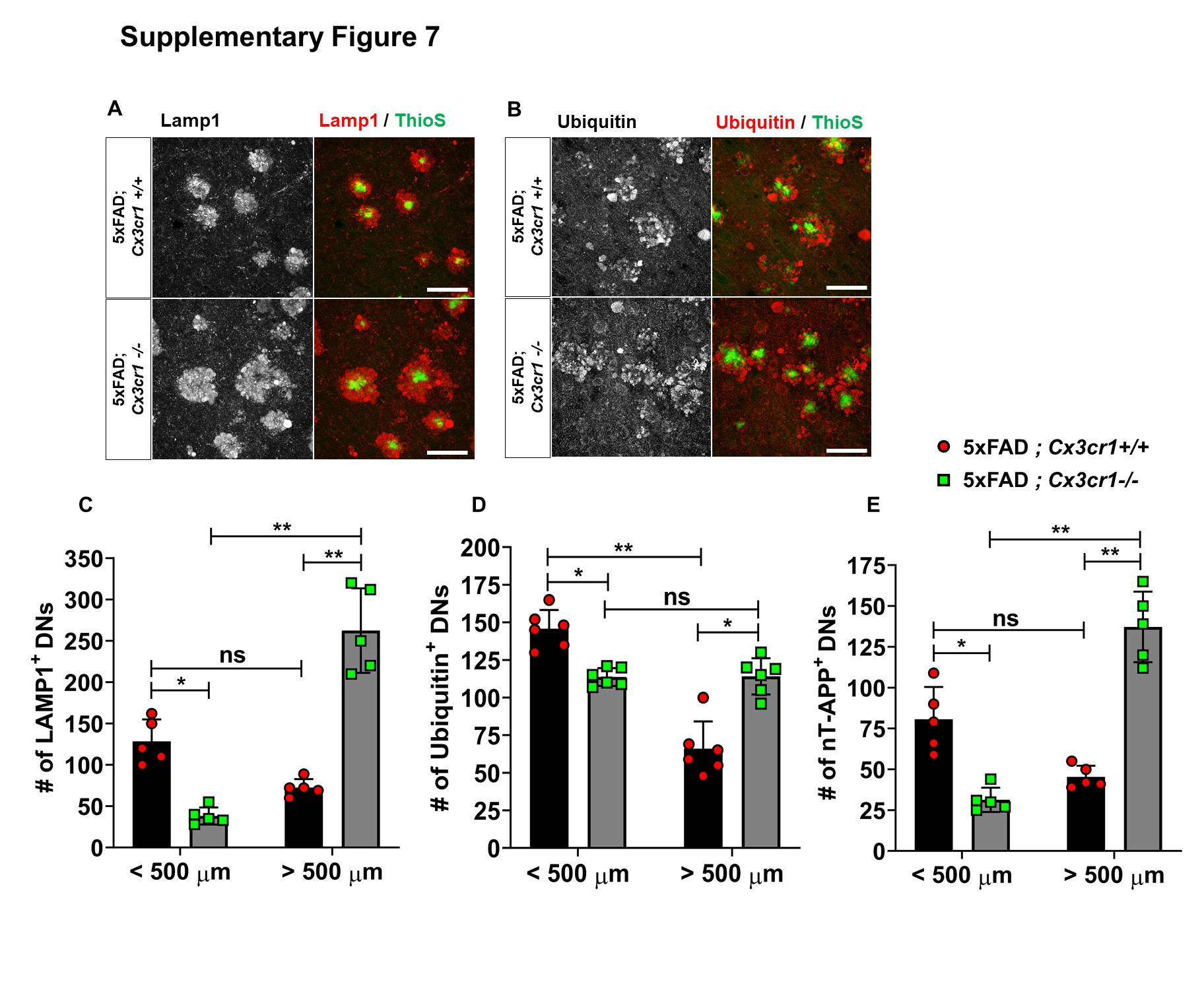

Supplement: Supplementary file 7 — Additional file 7: Supplemental Fig. 7. Larger dystrophic neurites (DNs) in 6 month-old 5xFAD;Cx3cr1-/- mice. Representative, high-resolution confocal images for (A) LAMP1+ DNs and (B) Ubiquitin+ DNs associated with ThioS+ plaques in 6-month old 5xFAD;Cx3cr1+/+ and 5xFAD;Cx3cr1-/- mice. Quantification of DNs in 6 month-old 5xFAD;Cx3cr1+/+ (black bars) and 5xFAD;Cx3cr1-/- (grey bars) using (A) α-LAMP1, (B) α-Ubiquitin and (C) α-nT-APP antibodies. Dystrophic neurites were quantified on the basis of their size, defined as small (<500µm; 50-500µm) vs. large (>500µm; 550-1000µm). Data in C-E represents mean cortical dystrophic neurite abundance calculated using multiple sections from n = 6 mice (3 females, 3 males). Statistical analysis done using Two way ANOVA (pint for LAMP1+ DNs, Ubiquitin+ DNs and nT-APP+ DNs <0.0001) followed by Tukey’s post-hoc tests. *p <0.05, **p < 0.005, ns – non-significant. [file 13024_2022_545_MOESM7_ESM.jpg]

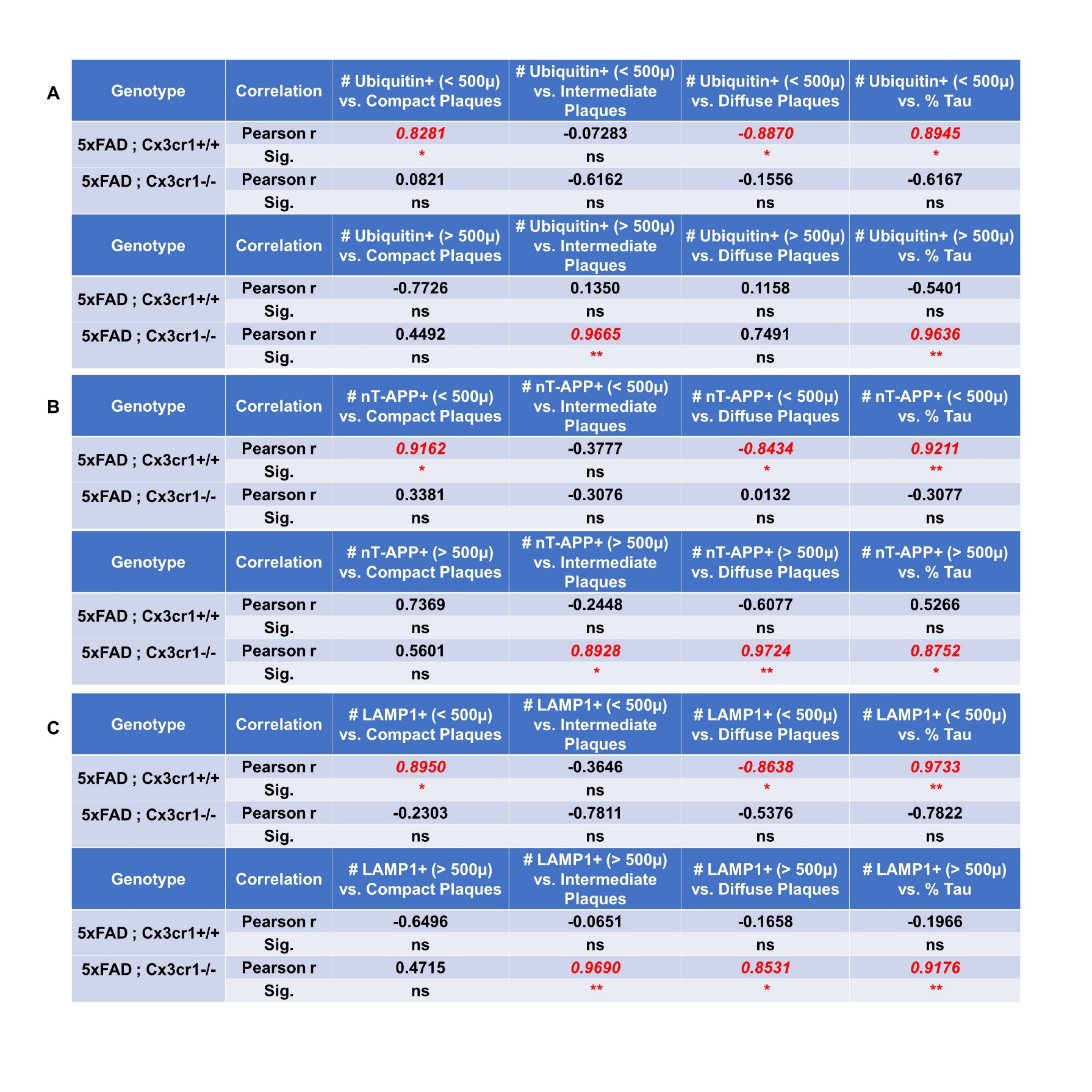
**Supplemental Table 2**

Supplement: Supplementary file 9 — Additional file 9: Supplemental Table 2. Pearson’s correlation analyses between cortical pTau pathology, small vs. large dystrophic neurites and plaque diffusivity in 6 month-old 5xFAD; Cx3cr1+/+ and 5xFAD; Cx3cr1-/- mice. Pearson’s correlation analyses were done using data from Supplemental Figure 7 (size distribution of DNs in cortex), Figure 1F (cortical proportions of compact, intermediate and diffuse plaques) and Figure 6C (%AT8+ cortical areas) to investigate interactions between small (<500µm) and large (>500µm) (A) Ubiquitin+, (B) nT-APP+ and (C) LAMP1+ dystrophic neurites and their relation to compact vs. intermediate vs. diffuse plaques and accumulation of AT8+ pTau in the cortices of 6 month-old 5xFAD; Cx3cr1+/+ and 5xFAD; Cx3cr1-/- mice. All data calculated using n = 6 (3 females, 3 males) of each genotype as described in materials and methods and related figure legends. Correlation matrices were calculated using imageJ. All data was tested for normality using the Anderson-Darling Test, and the Sharpiro-Wilk test for normality distribution. Significances were calculated using standard two-tailed t-tests with a 95% confidence interval. All significant interactions have been indicated in red. *p = 0.01, **p < 0.001, ns = not significant. [file 13024_2022_545_MOESM9_ESM.docx]
