# Supplementary material for: CX3CR1 deficiency aggravates amyloid driven neuronal pathology and cognitive decline in Alzheimer’s disease
Source: Mol Neurodegener. 2022 Jun 28;17:47. doi: 10.1186/s13024-022-00545-9 (PMC9241248; doi:10.1186/s13024-022-00545-9)
Supplement: Supplementary file 8 — Additional file 8: Supplemental Table 1. [file 13024_2022_545_MOESM8_ESM.docx]

**Supplemental Table 1.**

| ***Application*** | ***Antibody*** | ***Host Species*** | ***Vendor*** | ***Catalogue Number*** | ***Dilution / Concentration Used*** |
| --- | --- | --- | --- | --- | --- |
| **Histology**  DAB  Immunofluorescence  **Western Blot**  **ELISA** | Iba1 | Rabbit | Wako | 019-19741 | 1:1000 |
|  | Iba1 | Goat | Novus Biologicals | NB100-1028 | 1:1000 |
|  | Aβ42 (MOAB2) | Mouse (monoclonal) | Novus Biologicals | NBP2-13075 | 1:1000 |
|  | TREM2 | Sheep (polyclonal) | R&D Systems | BAF1729 | 1:500 |
|  | LAMP1 | Rat | Developmental Studies Hybridoma Bank | ID4B | 1:1000 |
|  | Ubiquitin | Rabbit | Fisher Scientific | PA1-10023 | 1:2000 |
|  | nT-APP | Mouse | EMD Millipore | MAB348 | 1:500 |
|  | AT8 | Mouse (monoclonal) | Thermo Fisher | MN1020 | 1:300 |
|  | AT180 | Mouse (monoclonal) | Thermo Fisher | MN1040 | 1:300 |
|  | NeuN | Mouse | Abcam | ab104224 | 1:3000 |
|  | Pu.1 | Mouse | Santa Cruz Biotechnology | sc-390405 | 1:200 |
|  | Ki-67 | Rabbit | Abcam |  | 1:200 |
|  | OC | Rabbit | Gift by Dr. Cristian Lasagna-Reeves | | 1:300 |
|  | Synaptophysin | Rabbit | Cell Signaling | 5461S | 1:20000 |
|  | SV2 | Rabbit | Synaptic Systems | 119-002 | 1:50000 |
|  | Homer | Rabbit | GeneTex | GTX103278 | 1:20000 |
|  | PSD95 | Mouse (monoclonal) | NeuroMab | 75-028 | 1:10000 |
|  | NMDAR1 | Mouse | Novus Biologicals | NB300-118 | 1:2000 |
|  | GAPDH | Rabbit | Cell Signaling Technology | 5174 | 1:10000 |
|  | GAPDH | Mouse | SantaCruz | sc-32233 | 1:10000 |
| **Flow Cytometry** | CD11b : Pe | Rat | Biolegend | 101202 | 1μl / 10^6^ cells |
|  | CD11b: PeCy7 | Rat | BD Biosciences | 561098 | 1μl / 10^6^ cells |
|  | CD45 : Pe | Rat | Biolegend | 103106 | 1μl / 10^6^ cells |
|  | CD45 : PeCy7 | Rat | BD Biosciences | 561868 | 1μl / 10^6^ cells |
