# Supplementary material for: CX3CR1 deficiency aggravates amyloid driven neuronal pathology and cognitive decline in Alzheimer’s disease
Source: Mol Neurodegener. 2022 Jun 28;17:47. doi: 10.1186/s13024-022-00545-9 (PMC9241248; doi:10.1186/s13024-022-00545-9)
Supplement: Supplementary file 10 — Additional file 10. Supplemental Materials and methods. [file 13024_2022_545_MOESM10_ESM.docx]

**Supplemental Materials and Methods:**

**Western Blotting:** Frozen cortical and hippocampal tissues were homogenized in ice-cold, T-Per Tissue Protein Extraction Buffer (ThermoFisher, Cat #78510) containing 1:100 phosphatase and 1:100 protease inhibitor cocktails. Following sonication, homogenates were centrifuged at 16,000xg, for 20 mins at 4ºC, clarified supernatants were transferred to Eppendorf tubes and stored at -80ºC. Protein concentrations were determined using the Bicinchoninic Acid (BCA) Assay Kit according to instructions (Thermo Fisher, Cat# 23225). Proteins were denatured at 95°C for 10 min in 3X denaturing buffer containing LDS sample buffer and 5% DTT. 15-25 μgs of protein per sample, along with 5 μls of SeeBlue Plus2 Prestained standard (ThermoFisher, Cat # LC5925) were loaded on to Novex 4–12% Bis-Tris gels (Life Technologies) and run at 120V for 90 mins in MES SDS running buffer (Novex ; Cat # NP0002). Proteins were transferred onto transferred onto PVDF membranes (EMD Millipore) in Tris-Glycine transfer buffer containing methanol at 100V for 2 hours on ice. Post-transfer, membranes were washed with Tris-buffered saline containing 0.1% Tween-20 (TBST), blocked with 5% BSA in TBST for 1 hour at RT and incubated with primary antibodies overnight at 4ºC. Membranes were washed in TBST and incubated with appropriate species-specific HRP-conjugated secondary antibodies for 1 hour at RT. Membranes were developed using the ImmobilonR Western Chemiluminescent HRP Substrate (EMD Millipore, Cat # WBKLS0500). ImageJ (National Institutes of Health) was used for densitometric analysis, and each sample was normalized to respective GAPDH. All primary antibodies used are listed in Supplementary Table 1.

**Brain Tissue Harvesting:** Mice were terminally anesthetized with intraperitoneal (i.p.) administration of a cocktail of Ketamine and Xylasene. Loss of pain perception was assessed based on the lack of a toe-pinch response. Following cardiac perfusion using ice-cold phosphate buffered saline (1XPBS), mice were decapitated, and the brain was divided into two halves along the sagittal midline. One half of the brain was post-fixed in ice-cold 4% paraformaldehyde (PFA) for 24-48 hours before further processing for immunohistochemical analyses. The remaining half was micro-dissected to separate anatomically distinct regions, namely the cortex and the hippocampus from the rest of the brain. Micro-dissected cortical and hippocampal tissue was stored at -80ºC for western blot analyses and RNA extraction used in transcriptomic analysis and qRT-PCR based gene expression studies. For flow cytometry studies, fresh brain tissue was collected in ice-cold 1X PBS immediately following cardiac perfusion.

**Tissue processing for flow-cytometry and FACS analysis:** Brain tissue from perfused cohorts of methoxy-X04 injected mice was enzymatically digested using the Miltenyi Neural Tissue Dissociation Kit (P) according to manufacturer’s instructions (Cat # 130-092-628). Brain homogenates were passed through 70µm nylon filters (Fisher Scientific, Cat # 22363548) to enable complete dissociation of plaque-associated microglia into single cell suspensions. Filtered homogenates were resuspended in RPMI supplemented with HEPES (SigmaAldrich, Cat # R7388), adjusted to 30% Percoll (GE Healthcare, Cat #17-0891-01) and underlaid with 1 ml of 70% Percoll. Following centrifugation at 800xg for 30 minutes at 4ºC, the cellular fraction at the 30% / 70% interface was harvested and viability of recovered cells was assessed trypan blue exclusion (Gibco, Cat # 15250061).

**Stereological quantification of microglial proliferation:** Serial sections from 6 month-old 5xFAD;*Cx3cr1^+/+^* and 5xFAD;*Cx3cr1^-/-^* mice (n=6, 3 females and 3 males) were stained with antibodies against Ki67, Pu.1 and Iba1 to identify proliferating microglial cells. Plaques were identified based on ThioflavinS staining. Plaques with intermediate (Circularity : 0.15-0.28) and diffuse (Circularity : 0.00 – 0.14) morphologies in cortical layer V were identified using ImageJ as described. Intermediate and diffuse plaques were chosen at random and were used for further analyses owing to significant increases in the proportion of these plaques in *Cx3cr1* deficient mice. Circular ROIs of 50µm diameter were drawn around identified cortical plaques, and plaque-associated total Iba1^+^DAPI^+^ microglia and Ki67^+^Iba1^+^DAPI^+^ microglia within these ROIs were quantified. 10,000-15,000 Iba1^+^DapI^+^ plaque-associated microglia were analyzed around ~300 plaques. ~ 500-600 non-plaque associated Iba1^+^DapI^+^ microglia were assessed for proliferation. All analyses were done using ImageJ.

**TREM2 ELISA:** F8 Maxisorp Nunc-Immuno Module (Thermo Fisher ; Cat 468667) wells were coated with 2µg/ml of the TREM2 capture antibody (R&D Systems ; MAB17291) in 0.05M carbonate/bicarbonate buffer (pH 9.6), overnight at 4°C, and blocked with 3% BSA, 0.05% Tween in PBS for 1 hour at RT. After blocking, cortical lysates diluted in ELISA buffer (0.5% BSA, 0.05% Tween in PBS) to 100ng/ul were incubated for 2 hours at RT. TREM2 standards were prepared using recombinant mouse TREM2 protein (R&D system ; Cat # 9228-T2). Following incubation for 2 hours at RT, plates were washed 4 times with 0.05% Tween in PBS and incubated with 0.25ug/ml of the TREM2 biotinylated detection antibody (R&D Systems ; Cat # BAF1729) for 1 hour at RT. After washing, samples were incubated with HRP-conjugated streptavidin (PerkinElmer ; Cat #NEL750001EA) diluted 1;10,000 in ELISA buffer. The samples were washed and incubated with the PierceTM TMB Substrate Mix (ThermoFisher ; Cat # 34021). Upon optimal color development, reactions were stopped using 1N HCL and plates were read at 450 nM using the Epoch2 microplate reader (BioTek).

**Behavioral Testing:** Changes in spatial working memory were assessed using the Y-maze. Briefly, following placement in the center of the maze, animals had free access to all three arms. In this paradigm, the mouse roams freely in all three arms of the Y-maze and can repeat entries in a single arm (re-entries), which results in a chance performance of 22.2% alternation. Thus, a spontaneous alternation score below 22.2% indicates a complete lack of working memory. Total number of arm entries and spontaneous alternations for each animal over an 8 minute period were recorded using the behavioral tracking software ANY-maze (Stoelting Co.). Spontaneous alternations were calculated using the formula (number of alternations)/((number of total entries)-2)x100. 6-10 male and female mice were used per genotype based on power analyses for an 80% probability of a 25% alternation in cognitive changes.

**Statistical Analyses:** Statistical analyses were done using the GraphPad Prism software. Standard, two-tailed student’s t-tests, with Welch’s correction for unequal standard deviations (SDs) were used for single comparisons between *B6;Cx3cr1^+/+^* vs. B6;*Cx3cr1^-/-^* and 5xFAD;*Cx3cr1^+/+^* vs. 5xFAD;*Cx3cr1^-/-^* cohorts. Two-way ANOVAs with recommended post-hoc tests and multiple comparisons were used for calculation of statistical differences between 5xFAD;*Cx3cr1^+/+^* and 5xFAD;*Cx3cr1^-/-^* cohorts, across 4 and 6 months of age, or for comparisons between small (<500µm; 50-500μm) vs. large dystrophic (>500µm; 550-1000μm) neurites. Significant interactions between factors were reported as p^int^. One-way ANOVAs with recommended post-hoc tests for multiple comparisons were used for calculating statistical differences between multiple testing cohorts for behavioral analysis, western blots and histological quantifications. Statistical differences were reported as adjusted p values (p^adj^). Data was graphed as mean +/- SEM to calculate error bars.
